# Temporal super-cell engineering and acoustic amplification in dispersive phononic time crystals

**DOI:** 10.1038/s41467-026-73459-5

**Published:** 2026-06-02

**Authors:** Ziling Liu, Xinghong Zhu, Zhi-Guo Zhang, Wei-Min Zhang, Xue Chen, Yong-Qiang Yang, Ruwen Peng, Mu Wang, Jensen Li, Hong-Wei Wu

**Affiliations:** 1 School of Mechanics and Photoelectric Physics, Center for Fundamental Physics, Anhui University of Science and Technology, Huainan, 232001 China; 2 Department of Physics, College of Science, Shantou University, Shantou, 515063 China; 3 Department of Physics, The Hong Kong University of Science and Technology, Clear Water Bay, Hong Kong, China; 4 National Laboratory of Solid State Microstructures, School of Physics, and Collaborative Innovation Center of Advanced Microstructures, Nanjing University, Nanjing, 210093 China; 5 Centre for Metamaterial Research and Innovation, Department of Physics and Astronomy, University of Exeter, Exeter, EX4 4QL United Kingdom

**Keywords:** Fluid dynamics, Acoustics

## Abstract

Floquet time crystals, characterized by momentum band gaps (*k*-gaps), offer powerful mechanisms for exotic wave control. However, selectively harnessing the Floquet band structure and opening multiple *k*-gaps remains a significant challenge in experiment. In this work, we construct a phononic time crystal by integrating discrete resonant meta-atoms into a one-dimensional acoustic waveguide, effectively creating a time-varying metamaterial. Through dynamic compressibility modulation, we observe amplified transmission and strong emission enhancement for a compact Floquet slab at the *k*-gap-associated frequency. Based on this versatile platform, we further extend the Floquet band physics by introducing a temporal-supercell concept that creates multiple *k*-gaps via momentum band folding. By suitably designing the compressibility in each phase of the supercell, we experimentally observe two clear amplified transmission frequency ranges around half and quarter of the original modulation frequency, for a corresponding compact Floquet slab with a band-folding-induced k-gap. This reconfigurable platform enables tailored parametric processes and unlocks pathways to higher-dimensional time crystals and topological temporal phenomena.

## Abstract (web summary)

Floquet time crystals allow exotic wave control through momentum band gaps (k-gaps), but experimentally harnessing their Floquet band structure and opening multiple k-gaps is difficult. Here, authors build a phononic time crystal using discrete resonant metaatoms as a compact Floquet slab, observe amplified transmission at a k-gap-associated frequency, and engineer multiple k-gaps via temporal supercells and momentum-space band folding.

## Introduction

Floquet time crystals constitute a new class of artificial materials, distinct from conventional spatial crystals. While spatial crystals are defined by periodic variations in space, Floquet time crystals feature constitutive parameters that remain uniform in space yet periodically modulated in time^1–5^. This temporal periodicity introduces discrete-time interfaces, leading to interference between time-reflected and time-refracted waves and giving rise to momentum band structures due to the breaking of discrete-time-translation symmetry. In contrast to the energy band gap in spatial crystals, Floquet time crystals exhibit momentum band gaps (k-gaps), which support two distinct Floquet modes: one grows exponentially and the other decays in time. Many intriguing phenomena have been theoretically predicted in photonic time crystals, including topological temporal edge states^6–9^, temporal Anderson localization^10,11^, amplified emission from electrons and dipole atoms^2,12^, and superluminal momentum-gap solitons^13^. To experimentally realize these effects, various time-varying photonic platforms have been developed. In the microwave regime, dynamic transmission lines have enabled the observation of k-gaps^14^, and temporally driven resonator arrays have revealed both Bloch-Floquet and non-Bloch band structures^15^. Additionally, time-varying metasurfaces have been employed to achieve exponential field growth within k-gaps by transforming volumetric systems into surface-based photonic time crystals^16^. Recently, the topological temporal boundary and the amplified wave in k-gap have also been observed in microwave^17,18^. However, realizing time-varying materials at optical frequencies remains a formidable task due to the requirement of ultrafast modulation, at least twice the optical carrier frequency. Promising candidates include all-optically modulated transparent conductive oxides, which offer significant changes in effective refractive index. Yet their application is hindered by the high optical pumping power required, which leads to thermal damage and limits performance^19–24^. Recent proposals have suggested expanding k-gaps using artificial resonators with time-varying resonance frequencies^25,26^, opening new directions for novel and more feasible modulation schemes.

As a universal concept, Floquet time crystals have been explored across various physical systems, including microwaves^16^, elastic waves^27–29^, water waves^30^, and acoustics^31^. However, realizing phononic time crystals for airborne sound remains a challenge, primarily because achieving fast, spatially uniform modulation of material properties is difficult. Previous strategies have included mechanically actuated resonators for nonreciprocal sound transmission^32^, yet these systems suffer from frictional losses, which limit both the achievable modulation rate and depth. Electroacoustic devices employing digital feedback have also been utilized to induce nonreciprocal mode transition^33^ by temporally switching the acoustic impedance of transducers, although they typically operate at low modulation frequencies. More recently, digitally virtualized meta-atoms have been introduced^34^, which consist of microphone and speaker pairs interconnected by an external microcontroller implementing a time-varying convolution kernel. Such platforms have enabled investigations into temporal effective medium theory^35,36^ at high modulation frequencies. Erewhile, two coupled cavities loading an external circuit are designed to observe the momentum-band topology in PT-symmetric Floquet lattices by temporally modulating the decay rate in acoustic^37^. Although recent experiments have sought to open a momentum-band gap in phononic time crystals, harnessing the momentum band structure and opening multiple *k*-gaps remain significant challenges in experiment, regardless of optical, acoustic, or elastic wave systems.

In this work, we experimentally realize a phononic time crystal by integrating programmable, time-varying resonant meta-atoms into a one-dimensional acoustic waveguide. By temporally modulating the resonant strength of these meta-atoms, we observe significantly amplified acoustic transmission, with a transmittivity exceeding 10, and emission enhancement, with a Purcell factor of 10 to a maximum of 30, for an inlaid monopole source within the k-gap-associated frequencies. Based on the versatile experimental platform, we further design temporal supercells that induce multiple Floquet band folding. The experiment results for amplified transmission indicate that the phononic time crystal with a temporal supercell not only exhibits an amplification at conventional k-gap-associated frequency around half the modulation frequency, but also a new amplification induced by band folding around a quarter of the original modulation frequency. Our well-designed time-varying metamaterials offer remarkable flexibility for engineering tailor-made dynamic responses and provide a powerful route for harnessing the Floquet band structure and enriching the physics of acoustic wave propagation.

## Results

### Constructing dispersive phononic time crystal

**Fig. 1:**
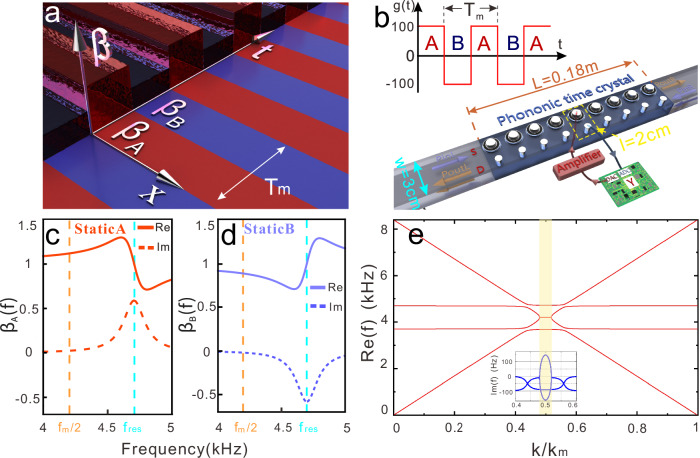
Concept of phononic time crystal and Floquet band structure. **a** Schematic picture of a phononic time crystal whose compressibility switches between *β*_A_ and *β*_B_ with period ${T}_{m}$. **b** Sketch map of an effective time-varying metamaterial constructed by 9 meta-atoms with a lattice length of 2cm, loading on a waveguide with a cross-section of 3cm × 3cm. Each meta-atom consists of a speaker (*S*) and a microphone (*D*) interconnected by a microcontroller (*Y*) performing a Lorentzian response. The resonating strength $g(t)$ is modulated between −100Hz to 100 Hz with period ${T}_{m}$. **c,** **d** Static compressibility for phase A and B, cyan dashed line represents the resonating frequency 4.7 kHz, brown dashed line corresponds to the half modulated frequency 4.2 Hz. **e** Floquet band structure of the phononic time crystal with time-varying compressibility switching between phase *β*_A_ and *β*_B_ in **c** and **d** with *k*_m_ = 153.9 rad/m. The insert gives the imaginary part of the Floquet frequency.

We begin with a one-dimensional (1D) phononic time crystal along the *x*-axis, as shown in Fig. 1a. Its compressibility $\beta (t)$, normalized by the compressibility of air ${\beta }_{0}$, is modulated over time (*t*) with a constant density ${\rho }_{0}$. For simplicity, we consider the temporal modulation with alternating phases A and B, over a modulation period *T*_*m*_, shown as red and blue stripes representing ${\beta }_{A}(f)$ and ${\beta }_{B}(f)$. Each phase occupies *T*_*m*_ /2. To experimentally realize the dispersive compressibility, we design 9 meta-atoms loading on a 1D waveguide with subwavelength lattice length $l=\,0.02\,m$ for constructing a homogenous metamaterial, as shown in Fig. 1b. Each meta-atom labeled by an index *i*, consists of a detector ${D}_{i}$ and a speaker ${S}_{i}$ arranged perpendicular to the waveguide with cross section $3{{\rm{cm}}}\times 3{{\rm{cm}}}$ for eliminating the spatial phase difference along the propagating direction. They are interconnected via a microcontroller that performs a time domain convolution on the detected signal. The resulting signal is feedback to the speaker to generate a resonating scattering response at each atom. The detailed operation in microcontroller for detected signal is presented in Note 1 of Supplementary Information. The meta-atom can mimic a time modulated resonator with a Lorentzian (named as positive Lorentzian)/ Anti-Lorentzian (named as negative Lorentzian) response in time. For example, the static Lorentzian-type compressibility can be described in the frequency domain as^35,36^:

1 $$\beta \left(f\right)\approx 1+\frac{{c}_{0}}{i\pi {fl}}Y(f),\,Y(f)=\frac{{ifg}}{{f}_{{res}}^{2}-{f}^{2}-2i\gamma f}$$

Here, the parameters are selected as: resonant strength $g=100\,{{\rm{Hz}}}$, resonant frequency ${f}_{{res}}=4.7\,{{\rm{kHz}}}$ and linewidth $\gamma=100\,{{\rm{Hz}}}$, ${c}_{0}=343\,{{\rm{m}}}/{{\rm{s}}}$ corresponds to sound speed in air. By modulating the resonating strength g$(t)$ between $-100{{\rm{Hz}}}$ and $100{{\rm{Hz}}}$ with duty cycle $\xi=0.5$ periodically in time (which can be seen in left-top insert of Fig. 1b), we can realize a temporal metamaterial with the compressibility $\beta \left(f\right)$ switching between two static configurations. The static compressibility configurations are shown in Fig. 1c and d, labeled as “Static A” and “Static B”, the transmission and reflection of a single static meta-atom with positive and negative responses are given in Supplementary Fig. 2.

Above, we have constructed a time-varying metamaterial that switches between positive and negative Lorentzian responses in time. Next, we discuss the Floquet band structure theoretically for a homogenous phononic time crystal. The airborne acoustic wave equations in waveguide are given as

2 $${\partial }_{x}p(x,t)+{\rho }_{0}{\partial }_{t}v\left(x,t\right)=0,$$

3 $${\partial }_{x}v(x,t)+{\beta }_{0}{\partial }_{t}\left(p(x,t)+M(x,t)\right)=0,$$

with $p(x,t)$, $v(x,t)$, $M(x,t)$ being pressure field, velocity field and the monopolar polarization, the monopolar response can be governed by a Lorentzian-type model

4 $${\partial }_{t}^{2}M(x,t)+2\varGamma {\partial }_{t}M(x,t)+{\omega }_{0}^{2}M(x,t)=a(t){\omega }_{0}^{2}p(x,t),$$

where ${\omega }_{0}=2{\pi }{f}_{{res}}$ is the resonating (radial) frequency, $\varGamma=2\pi \gamma$ is the resonating linewidth, $a\left(t\right)={c}_{0}g(t)/(\pi {f}_{{res}}^{2}l)$ being the resonance strength^36^. Under temporal modulation, the wave number $k$ remains unchanged across time interfaces. By substituting ${\partial }_{x}$ with ${ik}$, Eqs. (2), (3) and (4) can be written in matrix form as^36^

5 $$i{\partial }_{t}\psi=\hat{\omega }\psi,\,\hat{\omega }=\left(\begin{array}{cccc}0 & \frac{k}{{\beta }_{0}} & 0 & i\\ \frac{k}{{\rho }_{0}} & 0 & 0 & 0\\ 0 & 0 & 0 & -i\\ -{ia}\left(t\right){\omega }_{0}^{2} & 0 & i{\omega }_{0}^{2} & -i2\varGamma \end{array}\right),$$

with state vector $\psi={\left(p,v,M,-{\partial }_{t}M\right)}^{T}$. $\hat{\omega }$ is the propagation matrix. The state vector evolves as $\psi \left(t\right)={e}^{-i\hat{\omega }t}\psi \left(0\right)$. The Floquet modes satisfy $\psi \left(t+{T}_{m}\right)={e}^{-i{\hat{{{\rm{\omega }}}}}_{B}{T}_{m}/2}{e}^{-i{\hat{{{\rm{\omega }}}}}_{A}{T}_{m}/2}\psi \left(t\right)={e}^{-i\Omega {T}_{m}}\psi \left(t\right)$, with ${\hat{{{\rm{\omega }}}}}_{A}$/${\hat{{{\rm{\omega }}}}}_{B}$ being the propagation matrix in phase A/B and $\Omega=2\pi f$ being the Floquet frequency. This leads to the dispersion relation between $\Omega$ versus k by:

6 $$\det \left[{e}^{-i\Omega {T}_{m}}{I}_{4}-{e}^{-i{\hat{{{\rm{\omega }}}}}_{B}{T}_{m}/2}{e}^{-i{\hat{{{\rm{\omega }}}}}_{A}{T}_{m}/2}\right]=0,$$

where ${I}_{4}$ is the 4 by 4 identity matrix. Solving this secular equation in Eq. (6), Fig. 1e presents the band structure of a phononic crystal with dispersive compressibility modulated between ${\beta }_{A}(f)$ and ${\beta }_{B}(f)$ at a modulation frequency 1/*T*_*m*_ = 8.4 kHz. A characteristic wave number associated with the modulation frequency is defined as: ${k}_{m}=2\pi /({c}_{0}{T}_{m})$. Then, the band structure is plotted as ${{\rm{frequency}}}$ versus normalized wave number $k/{k}_{m}$, with red and blue lines denoting the real and imaginary parts of Floquet frequency, respectively. The k-gap region, highlighted in yellow, near half the modulation frequency (${f}_{m}/2=4.2{{\rm{kHz}}}$), corresponds to non-zero imaginary part of eigenfrequency, featuring one amplifying and one decaying Floquet mode. Additionally, four more quasi energy bandgaps^38^ are observed: two originate from the inherent resonance at 4.7 kHz, and the other two arise due to Floquet replica induced by time modulation, the detail is given in Supplementary Fig. 3. It should be pointed out that the material with the Lorentzian resonance here introduces an attenuation mode in the k-gap region. Unlike typical k-gap, the gap is no longer a “full” gap but a “partial” gap. This effect, however, does not suppress the temporal growth of the amplified Floquet mode.

### Experimental observation of amplified transmission in phononic time crystal

**Fig. 2:**
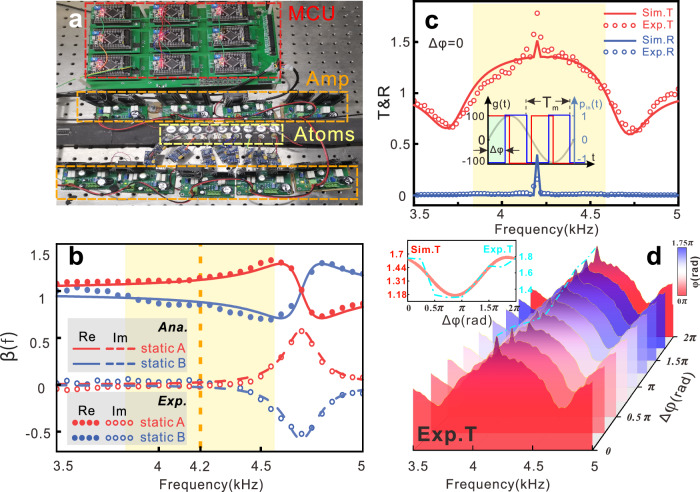
Experiment setup and amplified transmission. **a** Experimental platform of phononic time crystal with lattice distance $l$ = 0.02 m. **b** Static compressibility ${\beta }_{A}(f)$ (red line obtained from Eq. (1), red symbol extracted from experiment) in phase A and ${\beta }_{B}(f)$ (blue line is analytical result, blue symbol corresponds to experiment) in phase B. The parameters are chosen as same as Fig. 1: ${f}_{{res}}=4.7{{\rm{kHz}}}$, ${g}_{A}=100{{\rm{Hz}}}$, ${g}_{B}=-100{{\rm{Hz}}}$, $\gamma=100{{\rm{Hz}}}$. The faint yellow region is our interested frequency range around half modulation frequency 4.2 kHz marked by vertical orange dashed line. **c** Experimental and simulated transmittance and reflectance for phononic time crystal with modulated compressibility between A/B phases in **b** for $\varDelta \varphi=0$. It is defined in the inset as the zero phase difference between the incident wave arriving the leftmost metaatom represented by the gray sinusoidal curve and the red square wave modulation, the blue one has a positive phase delay $\varDelta \varphi$ comparing to the incident wave. **d** Transmission amplitude with respect to phase delay between incident wave and modulation cycle.

To experimentally observe amplification transmission in the k-gap-associated frequency as shown in Fig. 1 for demonstrating the capacity of our proposed platform, we construct an array of 9 meta-atoms in a 1D acoustic waveguide, which is same as Fig. 1b. We choose a chain of 9 meta-atoms with total length $L=0.18{{\rm{m}}}$ so that the amplified transmission strength induced by temporal modulation, which can be observed in the experiment. The details are discussed in Supplementary Fig. 4. The experimental set up is shown in Fig. 2a. As described as Fig. 1b, each meta-atom comprises a detector and a speaker (circled with yellow dashed box), the detector senses the acoustic pressure field in the waveguide and feeds the signal to the microcontroller (circled in red dashed box), which performs the atomic frequency response *Y*(*f*) but with possible time varying *g*(*t*) (as Note 1 of Supplementary Information). The resulting signal is then sent to the speaker through an amplifier (circled with an orange dashed box) to generate monopolar scattering, thereby realizing an effective compressibility $\beta (f)$ described by Eq. (1). We first evaluate the static compressibility of the two phases to verify ${\beta }_{A}(f)$ and ${\beta }_{B}(f)$ in the absence of time modulation. By scanning the incident frequency, we measure the transmission and reflection coefficients and extract the compressibility in the two phases^36^, as shown in Fig. 2b with red and blue symbols corresponding to the positive and negative Lorentzian-shape compressibility, respectively. Solid and hollow symbols represent the real and imaginary part. The experimental results show excellent agreement with the analytic results by Eq. (1) as the red and blue lines in Fig. 2b.

After establishing the static configurations, we modulate between two phases and measure the transmittance $T$ and reflectance $R$ spectra, presented in red and blue hollow dots in Fig. 2c. Since our meta-atoms involves feedback and time-varying resonant strength, the whole system (including all the atoms) is time dependent and frequency conversion occurs, thus we perform the stability analysis to make sure that the system is working in the stable regime as discussed in Note 4 of Supplementary Information. In the k-gap-associated frequency region, the transmittance is larger than 1, with larger value around half of the modulation frequency. The experiment results show great agreement with the simulation results, obtained from full-wave simulation using COSMOL Multiphysics (the simulation details are given in “Method”). Furthermore, two transmission valleys appears in the spectrum, at positions consistent with the expected quasi energy bandgaps of the band structure: one associated with the intrinsic resonating frequency 4.7 kHz and another due to the -1st harmonics 3.7 kHz for a modulation frequency of 8.4 kHz, as shown in Fig. 1e. To experimentally demonstrate that the amplified transmission originates from the interference between the Floquet modes, we also measure the Floquet mode components in the phononic time crystal when activing the temporal modulation. The result indicates that the outputting signals not only include the incident 0th order Floquet mode, but also have -1st order Floquet mode for tuning on the meta-atoms, as seen in Supplementary Fig. 6. Furthermore, we also measure the transmission and reflection spectra for different modulation frequency shown in of Supplementary Fig. 7. It is not difficult to find that the frequency range of amplified transmission shifts away from the original k-gap-associated frequency range marked by yellow region as the modulation frequency increases, and finally the transmission and reflection return to baseline (unity and zero) at higher modulation frequencies.

The central peak at half the modulation frequency ${f}_{m}/2$ arises from coupling between two Floquet components with 0th order and -1st order, depending on the phase difference $\varDelta \varphi$ as shown in the insert of Fig. 2c. To investigate this effect, we vary the phase difference $\varDelta \varphi$ between the incident signal and the modulation cycle from 0 to 2$\pi$ and remeasure the transmittance, plotted in Fig. 2d. The transmittance spectrum is largely phase insensitive except near ${f}_{m}/2$. Accordingly, we plot the transmittance at ${f}_{m}/2$ as a function of phase delay in the insert of Fig. 2d, using cyan dashed lines for experimental results and red lines for simulation. The transmittance goes through a cycle over 2$\pi$ and a minimum occurs when the incident wave is out of phase with modulation cycle. Despite the phase variation, the transmittance consistently remains greater than 1, demonstrating the robustness of the amplifying mode within the k-gap-associated frequency. We also note that this anomalous peak (the sharp peak at exactly *f*_m_/2) only emerges for continuous wave excitation, not pulsed wave excitation. In our phononic time crystal, the sample length is only about two wavelengths, so the bulk k-gap is not expected to be fully resolved in the finite structure. Nevertheless, parametric amplification around *f*_m_/2 remains significant in this compact Floquet slab. We further find that the amplified transmission increases with sample length and is well described by the finite-slab transmission formula ${t}={e^{{ik_{g}}{L}}}{{\rm{sec}}}(\Delta{k_g}L/2)$, where *k*_g_ and Δ*k*_g_ denote the centre and width of the k-gap, respectively. Although this expression is derived for a nondispersive Floquet slab surrounded by a constant medium of the same averaged compressibility (the free-space one in our case), it provides an effective description of our dispersive system when these quantities are obtained from the corresponding Floquet band structure.

### Emission enhancement in the k-gap-associated frequency

**Fig. 3:**
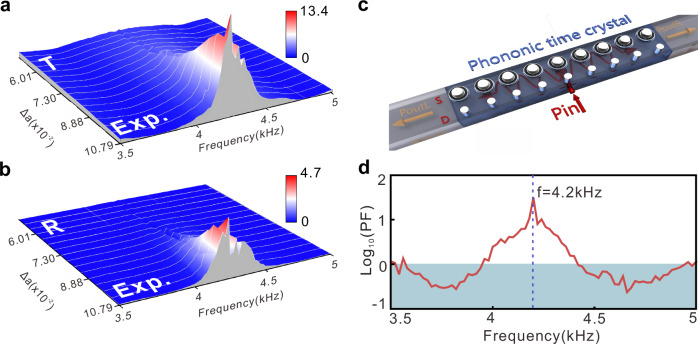
Amplified emission in k-gap-associated frequency. Transmittance **a** and reflectance **b** spectra with increased modulation depth Δ*a* in experiment. **c** Schematic picture for radiating source in phononic time crystal. **d** Purcell factor as a function of incident frequency.

In the previous section, we have demonstrated that our experiment platform can efficiently synthesize a phononic time crystal for temporally modulating between two static phases A and B. In Fig. 3a, we present the experimental transmittance as a function of modulating depth Δ*a* and frequency *f* for $\varDelta \varphi=0$. We observe that the transmittance increases from 1.7 to 13.4 as the modulating depth Δ*a* = $\frac{2{c}_{0}g}{\pi {f}_{{res}}^{2}l}$ is raised from 4.8 ×  10^-2^ to 10.79×10^-2^. Similarly, the reflectivity peak increases with increasing modulating depth at the k-gap-associated frequencies. In fact, these peaks are broader and more pronounced in experiment than in simulations, likely due to a small impedance mismatch between the realized system and the free-space and enhance residual backscattering in the finite sample.

It is well known that the emission enhancement is a unique property of Floquet time crystals^2,11^, whose eigenmodes grow temporally over time, independent of initial phase. To further demonstrate the amplified emission in the phononic time crystal, we place an acoustic source at the center of the phononic time crystal structure, as shown in Fig. 3c. To quantify the enhancement, we first deactivate all meta-atoms, and measure the outgoing sound pressure at both ends of the 1D empty waveguide, denoted as initial state: ${p}_{i}$. Next, we activate all meta-atoms to generate a k-gap-associated frequency around 4.2 kHz and record the amplified sound pressure, defining the final state: ${p}_{f}$. To characterize the amplified capacity of the phononic time crystal, we define the acoustic Purcell factor as ${PF}={\left|\frac{{p}_{f}}{{p}_{i}}\right|}^{2}$^39^, which is plotted in Fig. 3d as a function of incident frequency. We find that the *PF* can reach 10 on the broad peak to a maximum of 30 at 4.2 kHz for a modulating depth of Δ*a* = 10.79×10^-2^ under finite spatial length for continuous-wave excitation. In fact, the order of amplified emission can be further enhanced by increasing either modulating depth or the number of meta-atoms, leading to laser-like emission behavior.

### Floquet band folding and dual-band transmission amplifications by introducing temporal supercell

Until now, we have demonstrated the capability of well-designed meta-atoms for realizing the phononic time crystal by the experiments of amplified transmission and emission enhancement. In spatial phononic crystals, it is well known that expanding the unit cell to the supercell crystal causes the energy band structure to fold into the first Brillouin zone and open new bandgaps^40,41^.

**Fig. 4:**
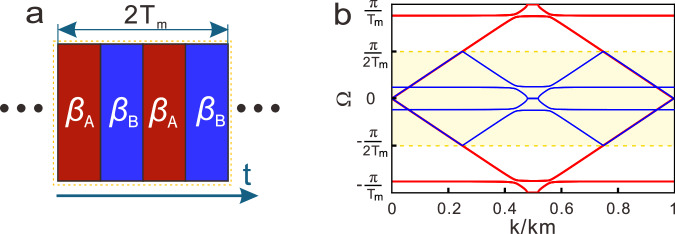
Temporal supercell and Floquet band folding. **a** Schematic diagram of temporal supercell with duration $2{{{\rm{T}}}}_{{{\rm{m}}}}$. **b** Floquet band structure of phononic time crystal with unit cell (red solid line) and supercell (blue solid line).

In the same spirit, we propose the Floquet band folding by expanding the unit cell “AB” shown in Fig. 1a to temporal supercell “ABAB” as shown in Fig. 4a. The material and modulating parameters are same as Fig. 1, except the temporal unit cell is expanded to supercell. The previous band structure ($\Omega$ verse *k*) relationship as Eq. (6) will be rewritten as: $\det \left[{e}^{-i\Omega 2{T}_{m}}{I}_{4}-{e}^{-i{\hat{{{\rm{\omega }}}}}_{B}{T}_{m}/2}{e}^{-i{\hat{{{\rm{\omega }}}}}_{A}{T}_{m}/2}{e}^{-i{\hat{{{\rm{\omega }}}}}_{B}{T}_{m}/2}{e}^{-i{\hat{{{\rm{\omega }}}}}_{A}{T}_{m}/2}\right]=0$ for the case of temporal supercell, and the resulting Floquet band structures are shown in Fig. 4b. Here, for the convenience of discussion, we calculate the Floquet frequency range from $-{{\rm{\pi }}}/{T}_{m}$ to ${{\rm{\pi }}}/{T}_{m}$ as vertical coordinates corresponding to the first Floquet Brillouin zone. The red bands correspond to the unfolded case for the temporal unit cell “AB”, with Floquet frequency periodicity $2{{\rm{\pi }}}/{T}_{m}$. After expanding the duration to ${2T}_{m}$, the red bands will be folded into the first Floquet Brillouin zone (yellow region), forming a blue band structure within the region $\pm {{\rm{\pi }}}/2{{{\rm{T}}}}_{{{\rm{m}}}}$ marked by yellow dashed lines. We can find that the primary k-gap is still around ${k}_{m}/2$ corresponding to *f*_m_/2, same as the red band structure. However, at ${k}_{m}/4$ and $3{k}_{m}/4$ corresponding to the frequencies of *f*_m_/4 and 3*f*_m_/4, the folding points are susceptible to perturbations (e.g., changes in material parameters, phase durations) and will open to form extra k-gaps.

**Fig. 5:**
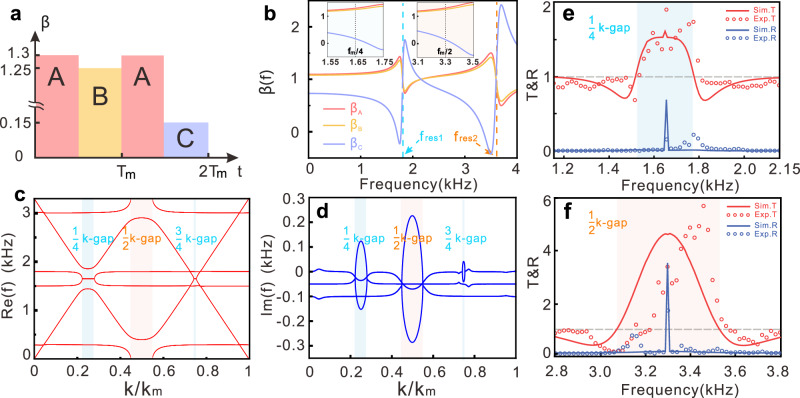
Floquet band folding and multiple transmission amplifications. **a** Conceptual picture of phononic time crystal with temporal supercell “ABAC”. The values of the vertical coordinate indicate the compressibility of static phases at $f={f}_{m}/4$ and ${f}_{m}/2$. **b** Static compressibility for different configurations A (*β*_A_), B (*β*_B_), C (*β*_C_) with two resonating responses, the vertical cyan and orange dashed lines marked the resonating frequencies ${f}_{{res}1}$ and ${f}_{{res}2}$, the top inserts shows the compressibility around the ${f}_{m}/4$ and ${f}_{m}/2$. **c,** **d** Band structure of phononic time crystal with temporal supercell in **a** for the real and imaginary part, the blue regions for both 1/4 k-gap and 3/4 k-gap, and the pink region correspond to the 1/2 k-gap. Experimental and simulated results of transmittance and reflectance spectra around the *f*_m_/4 **e** and *f*_m_/2 **f** region.

To open the folding points at ${k}_{m}/4$, here we introduce a perturbation in the material parameters (stack-ABAC) to construct phononic time crystal, as illustrated in Fig. 5a. The static phases A, B, C correspond to dispersive compressibilities *β*_A_, *β*_B_, and *β*_C_ as shown in Fig. 5b, respectively. Each phase lasts for ${T}_{m}/2$. For a practical experiment, we select the modulation frequency ${f}_{m}=6.6{{\rm{kHz}}}$ and the corresponding wave number ${k}_{m}=\frac{2\pi {f}_{m}}{{c}_{0}}=120.8{rad}/m$. The compressibility values of the three phases here also differ from those used in Fig. 1.

To experimentally observe the Floquet band folding and dual-band amplified transmissions, we design the meta-atoms with two resonances in $Y(f)$ of the Eq. (1), enabling an approximation of the same compressibility around ${f}_{m}/4$ and ${f}_{m}/2$, shown in top inserts of Fig. 5b. The resulting dispersive compressibility is given by:

7 $${\beta }_{n}\left(f\right)=1+\frac{{a}_{1n}{f}_{{res}1}^{2}}{{f}_{{res}1}^{2}-{f}^{2}-2i{\gamma }_{1}f}+\frac{{a}_{2n}{f}_{{res}2}^{2}}{{f}_{{res}2}^{2}-{f}^{2}-2i{\gamma }_{2}f},$$

where the subscript *n* represents static phases “A”, “B” and “C”, the resonating frequencies are ${f}_{{res}1}=$1.8 kHz$,\,{f}_{{res}2}=$3.6 kHz with decay rates ${\gamma }_{1}=50{{\rm{Hz}}},\,{\gamma }_{2}=100{{\rm{Hz}}}$. The resonating strengths are chosen to obtain the desired compressibility values ${\beta }_{A}=1.3$, ${\beta }_{B}=1.25$, ${\beta }_{C}=0.15$ at ${f}_{m}/4$ and ${f}_{m}/2$ as the schematic diagram in Fig. 5a. For example, to realize ${\beta }_{A}$, we set ${a}_{1A}=3{a}_{10},\,{a}_{2A}=3{a}_{20}$, as shown by the red line in Fig. 5b. The yellow line, representing static case B, corresponds to ${a}_{1B}=2.5{a}_{10},\,{a}_{2B}=2.5{a}_{20}$, while static case C as shown by the purple line requires ${a}_{1C}=-8.5{a}_{10},\,{a}_{2C}=-8.5{a}_{20}$, where ${a}_{10}=0.01345,\,{a}_{20}=0.0186$ corresponds to ${g}_{10}=7.98{{\rm{Hz}}},\,{g}_{20}=44.15{{\rm{Hz}}}$. To solve the band structure in this case, we can construct a 6 by 6 eigenvalue problem and solve the secular equation:

8 $$\det \left[{e}^{-2i\Omega {T}_{m}}{I}_{6}-{e}^{-i{\hat{{{\rm{\omega }}}}}_{C}{T}_{m}/2}{e}^{-i{\hat{{{\rm{\omega }}}}}_{A}{T}_{m}/2}{e}^{-i{\hat{{{\rm{\omega }}}}}_{B}{T}_{m}/2}{e}^{-i{\hat{{{\rm{\omega }}}}}_{A}{T}_{m}/2}\right]=0,$$

where ${I}_{6}$ is the 6 by 6 identity matrix, and the expressions of ${\hat{{{\rm{\omega }}}}}_{A}$, ${\hat{{{\rm{\omega }}}}}_{B}$, ${\hat{{{\rm{\omega }}}}}_{C}$ are given in Note 7 of Supplementary Information. Figures 5c and 5d present the real and imaginary parts of the Floquet band structure of the dispersive phononic time crystal under temporal modulation with the supercell of Fig. 5a. We observe that the band structure of the dispersive phononic time crystal firmly presents three k-gaps in ${k}_{m}/4$, ${k}_{m}/2$ and $3{k}_{m}/4$, denoted as $\frac{1}{4}$ k-gap, $\frac{1}{2}$ k-gap, and $\frac{3}{4}$ k-gap. The $\frac{1}{4}$ k-gap and $\frac{3}{4}$ k-gap come from the band folding and reopen as plotted in blue region, the $\frac{1}{4}$ k-gap has a gap width from $0.222{k}_{m}$ to $0.275{k}_{m}$ around $\frac{{k}_{m}}{4}$ corresponding to the frequencies from 1476Hz to 1842Hz around *f*_m_/4. For the chosen parameters in the designed compressibility, the $\frac{3}{4}$ k-gap at $k=0.75{k}_{m}$ is too narrow to have significant effect. In fact, if we design meta-atoms with three resonating responses to ensure same compressibility values around $\frac{{f}_{m}}{4}$, $\frac{{f}_{m}}{2}$ and $\frac{3{f}_{m}}{4}$, the $\frac{3}{4}$ k-gap width will be obviously expanded and the amplified transmission can also be measured in experiment. In this work, we mainly focus on the $\frac{1}{4}$ k-gap corresponding frequencies in the experiment due to the limitation of high-frequency in our waveguide. Furthermore, the width of $\frac{1}{4}$ k-gap depends on the compressibility contrast between the static phases B and C, the larger contrast, the wider the corresponding $\frac{1}{4}$ k-gap. The $\frac{1}{2}$ k-gap has a width from $0.446{k}_{m}$ to $0.548{k}_{m}$ as a pink region around $\frac{{k}_{m}}{2}$ relating to the associated frequencies from 2964Hz to 3624Hz around *f*_m_/2. Due to the high flexibility of our time-varying metamaterials in tailoring resonating responses as discussion in Note 1 of Supplementary Information, we experimentally implement the modulation for dispersive media with the compressibility shown in Fig. 5b at the phase difference $\Delta {{\rm{\varphi }}}=0$. Figure 5e shows the transmissivity and reflectivity at the frequency range around ${f}_{m}/4$ corresponding to the $\frac{1}{4}$ k-gap-associated frequency. Red lines and symbols represent simulated and experimental transmissivity, while blue lines and symbols denote the reflectivity results. The experiment results confirm the amplified transmission at the k-gap-associated frequency, as indicated by a transmission spectrum greater than 1. Furthermore, a similar high transmission spectrum is also observed at the second k-gap near ${f}_{m}/2$ corresponding to $\frac{1}{2}$ k-gap, as shown in Fig. 5f. The stronger transmission at $\scriptsize \it fm/2$ arises because the finite spatial length *L* = 0.18 m of phononic time crystal covers more modulation periods for higher operational frequencies. Furthermore, the amplified transmission peak of experimental measurement is slightly shifted compared with the simulated result in both k-gaps due to slight desynchronization of nine meta-atoms in the experiment. Comparing with $\frac{1}{4}$ k-gap-associated frequency, more obvious discrepancy for $\frac{1}{2}$ k-gap-associated frequency is that the inconsistence is magnified due to more temporal modulation periods for higher operational frequencies. In brief, our versatile time-varying metamaterials successfully demonstrate the band folding and multiple amplified transmissions through time modulation.

## Discussion

To summarize, we have experimentally demonstrated the emergence and control of Floquet band structure in phononic time crystals for airborne sound by implementing time-periodic modulation of compressibility. Utilizing acoustic meta-atoms with programmable, time-varying Lorentzian responses, we observed significant amplification in both acoustic transmission and emission within the *k*-gap-associated frequency. Building on this foundation, we introduced a temporal supercell with three distinct compressibility phases, enabling the formation of multiple Floquet band folding, as confirmed by transmission spectral measurements. We note that in our experiments, at the center of the *k*-gaps, an anomalous transmission peak appeared under continuous-wave excitation, whose amplitude exhibits stronger sensitivity than the broad peak to the phase difference between the incident wave and the modulation cycle. This anomalous peak, however, disappears under pulse-wave excitation.

In brief, the temporal supercell, in addition to non-periodic temporal modulation, such as the supercell definition in time quasicrystals^42^, provides a systematic route to engineer Floquet band structures in Floquet time crystals. By extending the modulation period, it enables predictable band folding and controllable k-gap opening, beyond ad hoc temporal modulation. The resulting k-gaps directly lead to sound-amplifying transmission, demonstrating nontrivial wave phenomena enabled by Floquet band engineering. Particularly, this control arises from hierarchical temporal modulation, where multiple periodicities introduced by the supercell provide structured degrees of freedom for independently tuning band folding, k-gap formation, and amplification. Such hierarchical control also enables more flexible engineering of Floquet band topology, while the temporal implementation allows in situ tuning within a single platform. Our platform with programmable meta-atoms makes this experimentally accessible. To our knowledge, temporal supercells have not been demonstrated experimentally, likely due to limited control over temporal modulation. Our system enables programmable spatiotemporal modulation and realization of both fundamental and supercell-induced k-gaps, providing a practical basis for exploring more complex band and topological effects.

## Methods

### Numerical simulation

In this work, the simulation is performed by a pressure acoustic model of COMSOL Multiphysics V6.3 in the time domain. The phononic time crystal is constructed using a lattice chain of meta-atoms in a one-dimensional waveguide. Each meta-atom (Fig. 1b) is modeled as a point source in its secondary radiation in the waveguide. The wave equations considering secondary radiation of the point sources are governed by:

$${\partial }_{x}p\left(x,t\right)+{\rho }_{0}{\partial }_{t}v\left(x,t\right)=0$$

9 $${\partial }_{x}v\left(x,t\right)+{\beta }_{0}{\partial }_{t}p\left(x,t\right)={\Sigma }_{i}\delta \left(x-{x}_{i}\right)\frac{2}{{\rho }_{0}{c}_{0}}{q}_{i}(t),$$

where ${c}_{0}=1/\sqrt{{\beta }_{0}{\rho }_{0}}$ is air sound speed, ${\rho }_{0}$ is the density of air and ${\beta }_{0}$ is the compressibility of air. The right-hand term of the equation represents the monopolar secondary point sources generated by speakers (${S}_{i}$) at positions ${x}_{i}$. For each atom, the Lorentzian model is implemented by a second order differential equation (ODE), which responds to the pressure field by

10 $${\partial }_{t}^{2}{q}_{i}\left(t\right)+2\Gamma {\partial }_{t}{q}_{i}\left(t\right)+{\omega }_{0}^{2}{q}_{i}(t)=-G(t){\partial }_{t}p\left({x}_{i},t\right),$$

where ${\omega }_{0}=2{\pi }{f}_{{res}}$ is the resonating (radial) frequency, $\Gamma=2\pi \,\gamma$ is the resonating linewidth, and $G(t)=2\pi g(t)$ is the resonance strength for each atom. ${q}_{i}$ has the same unit as the pressure $p$ in our convention. The $p({x}_{i},t)$ is the total pressure field picked by the detector ${D}_{i}$ which includes the incident field and the secondary radiation fields from all speakers. While resonating strength *g* can be generally time-varying to implement a specific compressibility of the metamaterial. In the harmonic representation of Eq. (10), the microcontroller implements a frequency response $Y$ (common for all atoms) defined by

11 $$Y\left(f\right)=\frac{{q}_{i}\left(f\right)}{p\left({x}_{i},f\right)}=\frac{{ifg}}{{f}_{{res}}^{2}-{f}^{2}-2i\gamma f}$$

The micro-controller only implements an open-circuit frequency response but in the weak scattering limit, such open-circuit frequency response approximates well the closed-circuit impulse response ${q}_{i}\left(f\right)/p\left({x}_{i},f\right)$. Then, in the homogenization limit, ${q}_{i}$ is dispersed into the lattice constant $l$ of the metamaterial, giving rise to $M={c}_{0}{q}_{i}/\left(i\pi {fl}\right)$. From the constitutive relationship $p+M=\beta p$, we can obtain the compressibility

12 $$\beta (f)=1+\frac{{c}_{0}Y}{i\pi {fl}}=1+\frac{a{f}_{{res}}^{2}}{{f}_{{res}}^{2}-{f}^{2}-2i\gamma f},$$

where $a={c}_{0}g/(\pi {f}_{{res}}^{2}l)$ is the overall resonance strength in the macroscopic level in specifying compressibility $\beta (f)$. Equations (9) and (10) are used to implement a specified Lorentzian resonance for $\beta (f)$. Based on the above, we can implement a time-varying metamaterial, e.g., in switching between two values of *a* (or $g(t)$) on the microscopic level, with a modulation period *T*_m_.

### Experimental detail

All experiments were conducted in a one-dimensional waveguide made of stainless steel tube, which featured nine virtualized atoms placed on top of it with a lattice constant of $l=$0.02 m, as depicted in Fig. 2. Each atom was composed of a speaker and microphone pair, with a microcontroller (Stm32f407rc) connected between them. The total delay time for each atom, comprising the delay time for the microphone, speaker, and electronic delay for the microcontroller, was experimentally obtained to be $\delta t=0.25$ ${{\rm{\mu }}}{{\rm{s}}}$ around 4.2 kHz (the half modulation frequency). To achieve a Lorentz resonant response with a digital microcontroller, Eq. (10) was transformed into a time-discrete model, but with the frequency response function $Y$ now approximated as the open-circuit frequency response function connecting ${D}_{i}$ to ${S}_{i}$ to be implemented electronically. The sampling frequency was set at 1.0 MHz, with a sampling period of 1 ${{\rm{\mu }}}{{\rm{s}}}$. Additionally, the program for the Lorentz response was designed to finish within one sampling period in preparation for the next sample to be sent to the speaker. The setup for a phononic time crystal composed of 9 atoms is depicted in Fig. 2. The 4-point measurement method is utilized in conjunction with a National Instruments DAQ device to determine the reflection and transmission signals. Through the application of the Fourier transform, we can obtain the transmission and reflection coefficients.

## Supplementary information

Supplementary Information

Transparent Peer Review file

## Data Availability

The theoretical and experimental data generated in this study have been deposited in the Baidu Netdisk database under accession code w869 [https://pan.baidu.com/s/1xJ-oIjpGAu7kqf1RZdG1NQ]. Other data that support the findings of this study are available from the corresponding authors upon request.
