# Supplementary material for: Temporal super-cell engineering and acoustic amplification in dispersive phononic time crystals
Source: Nat Commun. 2026 Jun 2;17:7079. doi: 10.1038/s41467-026-73459-5 (PMC13392367; doi:10.1038/s41467-026-73459-5)
Supplement: Supplementary file 1 — Supplementary Information [file 41467_2026_73459_MOESM1_ESM.pdf]

Supplementary Information for
"Temporal Super-cell Engineering and Acoustic Amplification in Dispersive Phononic Time Crystals"

Ziling Liu[1†], Xinghong Zhu[2, 3†], Zhi-Guo Zhang[1†], Wei-Min Zhang[1], Xue Chen[1], Yong-Qiang Yang[1], Ruwen Peng[4*], Mu Wang[4*], Jensen Li[5*], and Hong-Wei Wu[1, 4*]

[1]School of Mechanics and Photoelectric Physics, Center for Fundamental Physics, Anhui University of Science and Technology, Huainan 232001, China

[2]Department of Physics, College of Science, Shantou University, Shantou 515063, China

[3]Department of Physics, The Hong Kong University of Science and Technology, Clear Water Bay, Hong Kong, China

[4]National Laboratory of Solid State Microstructures, School of Physics, and Collaborative Innovation Center of Advanced Microstructures, Nanjing University, Nanjing 210093, China

[5]Centre for Metamaterial Research and Innovation, Department of Physics and Astronomy, University of Exeter, Exeter EX4 4QL, United Kingdom

Supplementary Note 1: Performed operation in microcontroller for meta-atoms

Supplementary Fig. 1 shows a single atom in a 1D airborne acoustic waveguide consisting of a detector and a speaker (labeled in D and S) interconnected by a microcontroller. The signals detected by the microphone D convolute with Y in time domain in the microcontroller and then feedback to the speaker S to generate a monopolar radiation:

$$S(t) = \int_0^t Y(t')D(t-t')dt'. \tag{S1}$$

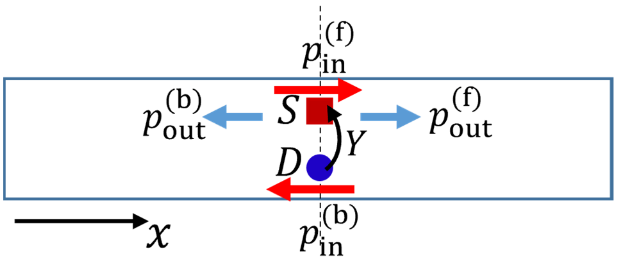

Supplementary Fig. 1. Schematic of a single atom in the acoustic waveguide.

The linear operation between the sound pressure at D and S in frequency domain can be described as:

$$S(f) = Y(f)D(f) \tag{S2}$$

Within the waveguide, the radiation from S combines with the forward (backward) incident pressure $p_{\text{in}}^{(\text{f})}$ and $p_{\text{in}}^{(\text{b})}$ to become the pressure field at D by

$$D(\omega) = p_{\text{in}}^{(\text{f})} + p_{\text{in}}^{(\text{b})} + S(f) \tag{S3}$$

The sound pressure on the right side ($p_{\text{out}}^{(\text{f})}$) and on the left side ($p_{\text{out}}^{(\text{b})}$) can be written as

$$p_{\text{out}}^{(\text{f})} = p_{\text{in}}^{(\text{f})} + S(f), \qquad p_{\text{out}}^{(\text{b})} = p_{\text{in}}^{(\text{b})} + S(f). \tag{S4}$$

Combing Eqs. S(1) to S(3) gives the scattering response of the meta-atom as

$$\begin{pmatrix} p_{\text{out}}^{(\text{f})} \\ p_{\text{out}}^{(\text{b})} \end{pmatrix} = \begin{pmatrix} t & r \\ r & t \end{pmatrix} \begin{pmatrix} p_{\text{in}}^{(\text{f})} \\ p_{\text{in}}^{(\text{b})} \end{pmatrix} \tag{S5}$$

with the reflection and transmission coefficient

$$r = \frac{Y(f)}{1 - Y(f)}. \tag{S6}$$

In the main text, we set

$$Y(f) = \frac{ifg}{f_{res}^2 - f^2 - 2i\gamma f}, \tag{S7}$$

where g, f_{res} and γ are the resonant strength, resonant frequency and linewidth. In weakly scattering regime (Born approximation), we have

$$\frac{r}{i} \cong \frac{Y(f)}{i} = \frac{fg}{f_{res}^2 - f^2 - 2i\gamma f}. \tag{S8}$$

It can mimic a Helmholtz resonator with resonant frequencyf_{res}, resonant strength g and linewidth γ. In the actual program, the Fourier transform has to be turned into z-transform in processing discrete time signal:

$$S(z) = Y(z)D(z) = \frac{Y^{(n)}(z)}{1 + Y^{(d)}(z)} D(z), \tag{S9}$$

where $Y^{(n)}(z)$ and $Y^{(d)}(z)$ (without constant term) are polynomials of z^{-1} with a finite number of terms while the Fourier transformed signal can be obtained by substituting $z = exp(-ifT_s)$ with T_s being the sampling period of the signal by the microcontroller so that $Y(f) \cong Y(z)$ where $z \cong (1 - ifT_s/2)/(1 + ifT_s/2)$. In the implementation, we use the following "program" to update the current sample $S(n)$ by

$$S(n) + c_1 S(n-1) + c_2 S(n-2) \tag{S10}$$

$$= b_0 D(n) + b_1 D(n-1) + b_2 D(n-2),$$

where

$$\begin{aligned} Y^{(n)}(z) &= (b_0 + b_1 z^{-1} + b_2 z^{-2}), \\ Y^{(d)}(z) &= (c_1 z^{-1} + c_2 z^{-2}). \end{aligned} \quad \text{(S11)}$$

To obtain a time-varying meta-atom, we only need to flip the sign of the coefficient of b_0, b_1 and b_2 in the program, effectively modulating the resonant strength g(t).

To provide insight into how the system behaves under temporal modulation, we also give the transmission and reflection of single static meta-atom with positive and negative Lorentzian response as shown in Supplementary Fig. 2. The resonating frequency is defined as 4.7kHz. The red lines and symbols represent the analytical and experimental transmission (T), the blue lines and symbols correspond to the analytical and experimental reflection (R). The yellow region indicates the amplified transmission region when temporal modulation with modulating frequency 8.4kHz between the two static cases.

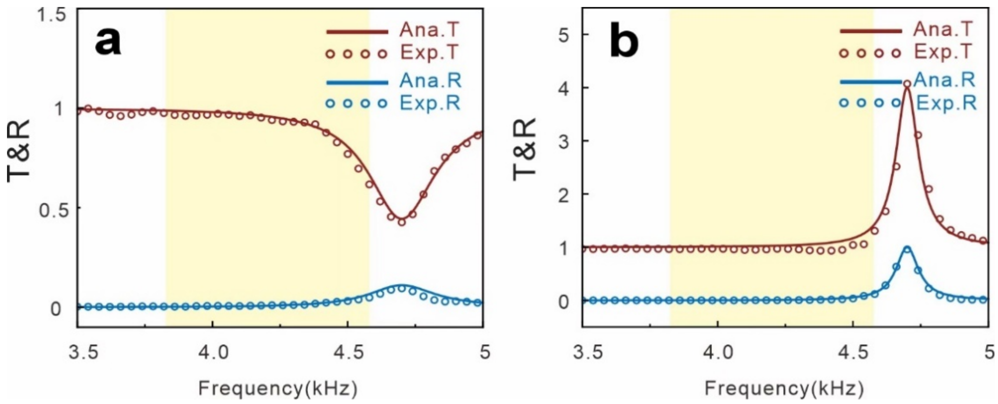

Supplementary Fig. 2. The transmission (T) and reflection (R) of single static meta-atom with **a** positive and **b** negative Lorentzian response.

Supplementary Note 2: Band structure for time-varying dispersive media with Lorentzian-type resonance

To further clarify the Floquet band structure of phononic time crystal in Fig. 1 of main text, we decompose the temporal modulation between positive and negative Lorentzian response into unilateral modulation. In Supplementary Fig. 3, we firstly give the band structure of phononic time crystals with modulated resonating strength $a_A = 0.025$ and $a_B = -0.025$, whose real and imaginary part shown in Supplementary Figs. 3a and 3b. Except the k-gap around $k = k_m/2$, there are extra four gaps presented in

Supplementary Fig. 3a marked as “A”, “B”, “C”, “D”.

To clearly clarifying the formation of these gaps, we calculate the Floquet band structure for modulating the resonating strength of positive Lorentzian response singly. Supplementary Figs. 3c1 and 3c2 show the real part and imaginary part for static positive Lorentzian response with $a_A = 0.025$ and $a_B = 0.025$. The real part of two-band complex band structure as red lines present the up branch “$\mathrm{F}_{\mathrm{up}}^{0}$” and low branch “$\mathrm{F}_{\mathrm{lo}}^{0}$” corresponding to the 0 order Floquet mode due to the Lorentzian response with resonating frequency 4.7 kHz. The grey bands describe the upcoming location of the -1st order Floquet mode when activating the temporal modulation with $\mathrm{T_m} = 1/8.4\mathrm{kHz}$. The imaginary part as blue line show a cross at $k = 0.56k_m$ represent transform between the localized mode and propagated mode. When we activate the temporal modulation between $a_A = 0.025$ and $a_B = 0.0$ with modulating frequency 8.4kHz, the k-gap is open and a Floquet amplified mode presented as imaginary part in Supplementary Fig. 3d2, which comes from the interference between the Floquet modes of “$\mathrm{F}_{\mathrm{lo}}^{0}$” and “$\mathrm{F}_{\mathrm{up}}^{-1}$”. Furthermore, two gaps located at $(0.56k_m, 4.7\mathrm{kHz})$ and $(0.56k_m, 3.7\mathrm{kHz})$ correspond to the resonation “A” and Floquet replica “B” due to the temporal modulation. At $k = 0.44k_m$, the interaction between Floquet modes “$\mathrm{F}_{\mathrm{up}}^{-1}$” and “$\mathrm{F}_{\mathrm{up}}^{0}$” are weak and the gap not be opened, which can also be seen in the blue line of Supplementary Fig. 3d2. Accordingly, the case between Floquet modes “$\mathrm{F}_{\mathrm{lo}}^{-1}$” and “$\mathrm{F}_{\mathrm{lo}}^{0}$” is similar. When we increase the modulating depth in Supplementary Fig. 2e1, comparing with Fig. S3d1, the opened k-gap and gaps “A” and “B” will further enlarge. Otherwise, the cross point at $k = 0.44k_m$ as red line will also be opened for forming the gaps “C” and “D”, and the blue line presents a cross point.

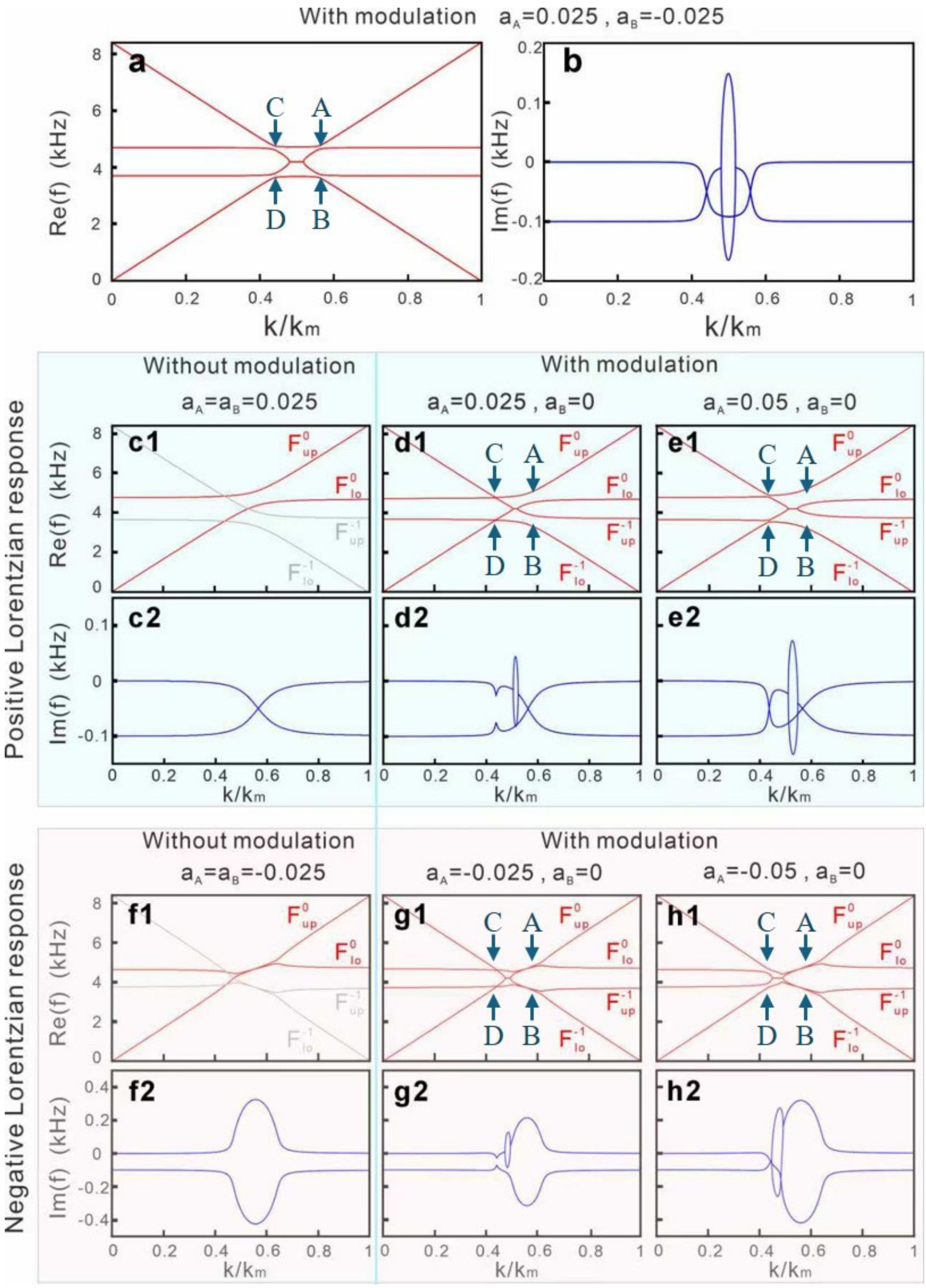

Supplementary Fig. 3. The real **a** and imaginary **b** part of the complex band structure for temporal modulating the resonating strength between $a_A = 0.025$ and $a_B = -0.025$. The arrows "A", "B", "C" and "D" are the extra four gaps. **c1** and **c2** correspond to the complex band structure of static positive Lorentzian response for $a_A = 0.025$ and $a_B = 0.025$. **d1** and **d2** for $a_A = 0.025$ and $a_B = 0.0$. **e1** and **e2** for $a_A = 0.05$ and $a_B = 0.0$. **f1** and **f2** for $a_A = -0.025$ and $a_B = -0.025$. When activating the modulation for $a_A = -0.025$ and $a_B = 0.0$, the complex band

structure is shown in **g1** (real part) and **g2** (imaginary part). Band structure in **h1** and **h2** corresponds to $a_A = -0.05$ and $a_B = 0.0$.

At the other hand, we also calculate the complex band structure for the compressibility with negative Lorentzian response. Supplementary Figs. 3f1 and 3f2 give the real and imaginary part as red line and blue line for $a_A = -0.025$ and $a_B = -0.025$. Different with positive Lorentzian response, the real part of band structure showcase a merging point between Floquet modes "$\mathrm{F}_{\mathrm{lo}}^{0}$" and "$\mathrm{F}_{\mathrm{up}}^{0}$", rather than avoided crossing point. The upcoming -1 order Floquet modes are described as grey line "$\mathrm{F}_{\mathrm{lo}}^{-1}$" and "$\mathrm{F}_{\mathrm{up}}^{-1}$". The imaginary part presents a gain and a loss branch in Supplementary Fig. 3f2. When we activate the modulation of resonating strength between $a_A = -0.025$ and $a_B = 0.0$, the modes "$\mathrm{F}_{\mathrm{up}}^{-1}$" and "$\mathrm{F}_{\mathrm{lo}}^{0}$" will interfere each other and form k-gap around $k = k_m/2$ as shown in Supplementary Fig. 3g1, the circle presents in Supplementary Fig. 3g2. The arrow locations "A" and "B" indicate the merging points. When larger increasing the modulating strength to $a_A = -0.05$ and $a_B = 0.0$, the k-gap further enlarges, and the cross points at "C" and "D" are open as shown in Supplementary Fig. 3h1.

United the imaginary parts of Supplementary Figs. 3d2 and 3g2 together, we can find that the circle of imaginary part comes from the temporal modulation, rather than the resonating response. Particularly, the band structures presented in Supplementary Fig. 3a and Supplementary Fig. 3b are a synergistic effect from the modulating positive and negative Lorentzian response, for example, the larger k-gap around $k = 0.5k_m$ comes from the superposition between Supplementary Fig. 3d1 and Supplementary Fig. 3g1. At $k = 0.44k_m$, the two cross points are pull open due to the superposition effect for forming the gaps as marked by "C" and "D" of Supplementary Fig. 3a. Nevertheless, at $k = 0.56k_m$, the avoided crossing "A", "B" of Supplementary Fig. 3d1 and the merging point "A", "B" of Supplementary Fig. 3g1 will neutralize each other for forming the gaps "A" and "B" in Supplementary Fig. 3a. These synergistic effects construct the symmetric complex band structure around $k = 0.5k_m$, which hold a k-gap and four ω-gaps denoted by "A"-"D".

Supplementary Note 3: The influence of meta-atom number on the transmission and reflection of phononic time crystal

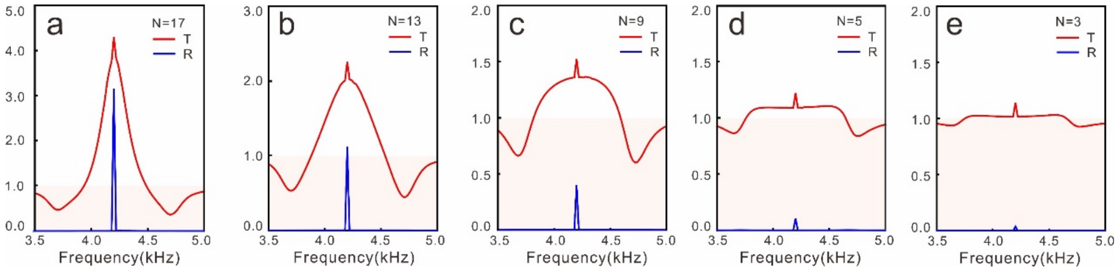

Supplementary Fig. 4. Transmittance (T) and reflectance (R) of 1D phononic time crystals for different meta-atom numbers N = 17, 13, 9, 5, 3.

To demonstrate that the 9 meta-atoms in our work are sufficient to observe amplified transmission in experiment, we calculate the transmittance (T) and reflectance (R) of phononic time crystals with different meta-atom numbers N = 17, 13, 9, 5. We can find that the transmission and reflection strength in the k-gap depends on the meta-atom number, and the phononic time crystal with more meta-atoms presents more obvious T&R as shown in Supplementary Fig. 4. Considering the practical experiment, we construct 9 meta-atoms in our work, the T&R in Supplementary Fig. 4c clearly presents the amplified transmission, the two transmission dips located at 4.7kHz and 3.7kHz correspond to the ω-gaps in the momentum band structure due to inherent resonance and Floquet replica. Though the T and R is weak comparing with the case of more meta-atom numbers, while the amplified T is sufficient to observe in experiment for demonstrating the parametric amplification under temporal modulation.

Supplementary Note 4: Stability analysis for phononic time crystals in the experiment

Since our meta-atoms involves feedback and time-varying resonant strength, the whole system (including all the atoms) is time dependent and frequency conversion occurs, we need stability analysis to make sure that the system is working in the stable regime. In this section, we develop a multiband harmonic model to determine the eigenmode (stability) of the time-dependent system. As the modulation strength and resonating frequency is modulated periodically, all the signals (from the detector and speaker) can be written in the Fourier series expansion with a set of harmonics $f_n = f + nf_m$, where n is an integer and f_m is the modulation frequency. The detector and speaker signal can be expanded as $D(x_i, t) = \sum_n p_{in} e^{-if_n t}$, $S_i(t) = \sum_n s_{in} e^{-if_n t}$ where i

represents the i-th atom. The modulation of resonating strength and resonating frequency can be written as $g(t) = \sum_n g_n e^{-int/T_m}$,where $f_{res} = 4.7$kHz. Then, the response function Y can be written in terms of Fourier component (labeled by n harmonics): $Y^i_{nn'} = \frac{if_n g_{n-n'}}{f_{\text{res}}^2 - f_n^2 - 2i\gamma f_n}$ and it connects the detector and speaker signal by $S_{in} = Y^i_{nn'} D_{in'}$. Since the convolution generates the secondary radiation and propagates in waveguide and then is detected again, there is a loop transfer function from detector D_i to D_j by $\mathcal{T}_{\{jn\},\{in'\}}(\omega) = e^{ik_0(f_n)|x_j - x_i|} Y^i_{nn'} = e^{ik_0(f_n)|x_j - x_i|} \frac{if_n g_{n-n'}}{f_{\text{res}}^2 - f_n^2 - 2i\gamma f_n}$ where $|x_j - x_i|$ is the distance from speaker i to detector j (assuming D_i and S_i are in the same position since they are collocated). The $\mathcal{T}_{\{jn\},\{in'\}}$ is a square matrix and a stable system means all the poles the of the system response matrix, i.e. zeros of $Det(I - \mathcal{T}_{\{jn\},\{in'\}})$ have to lie in the lower complex plane. The number of harmonics (a total of 7 in the following analysis) is taken to be enough to have converging numerical results. For the time-varying acoustic system in Fig. 2 (modulating resonant strength $g(t)$ between positive and negative to generate modulating depth 200Hz), we plot the $Det(I - \mathcal{T}_{\{jn\},\{in'\}})$ in the complex frequency plane, as shown in Fig. S5a. The minimum value of $Det(I - \mathcal{T}_{\{jn\},\{in'\}})$ in the blue dot indicates the pole of the system, around the frequency of (4690-i140)Hz, in the lower half plane. This means the system is working in the stable regime. When we increase the modulation depth, the system poles will gradually touch the real frequency axes and make the system become unstable. Supplementary Fig. 5 plots the pole diagram where one of the poles just touch the real frequency axes, corresponding to the maximum modulation depth $\Delta g = 14.56 * 200\text{Hz}$ (Δa =31.05×10^{-2}). However, in the experiment, we only obtain the threshold at Δa =10.79×10^{-2} due to the intrinsic response of speaker and time delay of microcontroller making the system easier to become unstable.

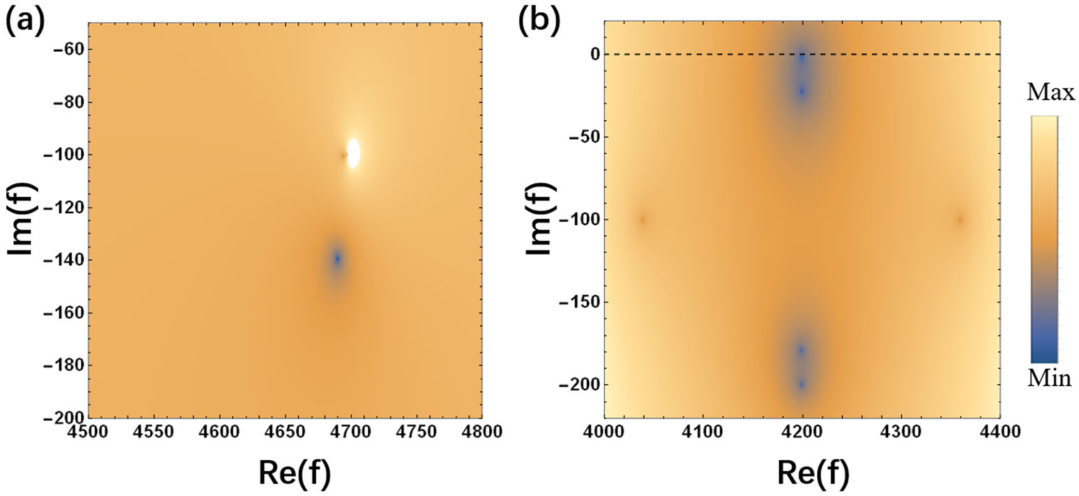

Supplementary Fig. 5. a Pole diagram of time-varying meta-atoms in Fig.2, **b** Pole diagram of time-varying meta-atoms with threshold touching the real axes.

Supplementary Note 5: Floquet mode components in phononic time crystal

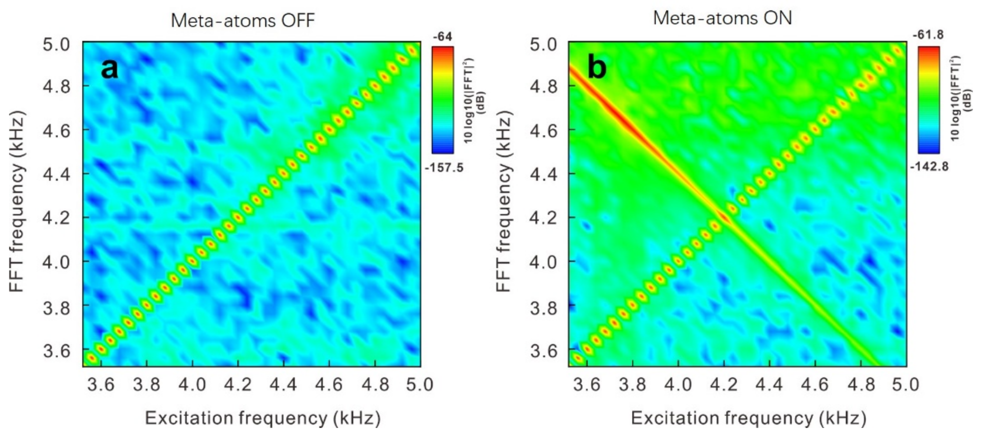

Supplementary Fig. 6. The outputting signal analysis for incident signal with frequency from 3.5kHz to 5.0kHz for **a** closing meta-atoms (air waveguide) and **b** opening meta-atoms (phononic time crystal).

To demonstrate the amplified transmission for temporal modulating resonating strength in experiment, we do fast Fourier transform (FFT) algorithm for the outputting signal when the frequency of inputting signal scanning from 3.5kHz to 5.0kHz. We can find that the outputting signal with same frequency as the inputting signal for closing the meta-atoms, i.e., this is a test experiment for air waveguide as shown in Supplementary Fig. 6a. However, when we open all meta-atoms to temporally modulate the resonating strength $g(t)$ between 100Hz and -100Hz with modulating frequency 8.4kHz, it is not difficult to find that the -1st order Floquet mode will be generated together with the 0 order Floquet mode, and cross at half modulating frequency 4.2kHz,

as can be seen in Supplementary Fig. 6b. The result also demonstrates the statement that the amplification comes from the Floquet band folding and interference between the Floquet modes of "F_{lo}^{0}" and "F_{up}^{-1}".

Supplementary Note 6: The influence of modulating frequency on the amplified transmission

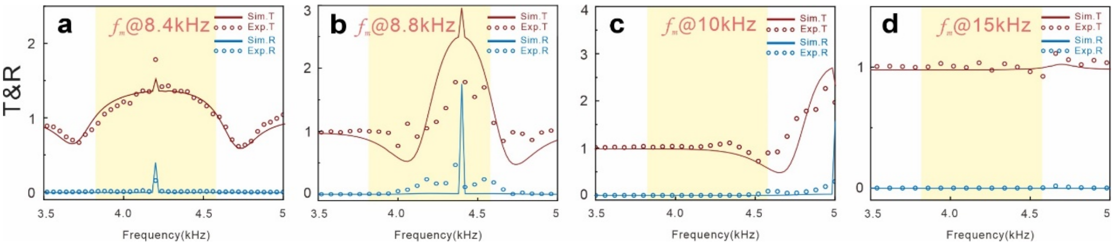

Supplementary Fig. 7. The transmission and reflection of 1D phononic time crystal for different modulating frequency **a** 8.4kHz, **b** 8.8kHz, **c** 10kHz, **d** 15kHz by simulation and experiment.

To further demonstrate that the amplified transmission originates in the temporal modulation, we increase the modulating frequency from 8.4kHz to 15kHz and keep the resonating frequency at 4.7kHz as Supplementary Fig. 7. It is not difficult to find that the amplified transmission region will moves away from original frequency range marked by yellow region to higher frequency, and finally the transmission and reflection restore calm in primary yellow region for $f_m = 15\text{kHz}$.

Supplementary Note 7: Band structure for dispersive media with two Lorentzian-type resonances

Following the same spirit of route, when we have two monopolar resonances in each phase, we only need to add another response function into the wave equation as

$$\partial_x v(x,t) + \beta_0 \partial_t (p(x,t) + M_i(x,t)) = 0, \tag{S12}$$

$$\partial_t^2 M_i(x,t) + 2\Gamma_i \partial_t M_i(x,t) + \omega_{0i}^2 M(x,t) = a_i(t)\omega_{0i}^2 p(x,t), \tag{S13}$$

with $i = 1, 2$, representing the first and second resonance mode. Combined with Eq. (2) in main text, we can construct a 6 by 6 eigenvalue problem as

$$i\partial_t \psi = \hat{\omega}\psi, \hat{\omega} = \begin{pmatrix} 0 & k/\beta_0 & 0 & i & 0 & i \\ k/\rho_0 & 0 & 0 & 0 & 0 & 0 \\ 0 & 0 & 0 & -i & 0 & 0 \\ -ia_1(t)\omega_{01}^2 & 0 & i\omega_{01}^2 & -2i\Gamma_1 & 0 & 0 \\ 0 & 0 & 0 & 0 & 0 & -i \\ -ia_2(t)\omega_{02}^2 & 0 & 0 & 0 & i\omega_{02}^2 & -2i\Gamma_2 \end{pmatrix} \tag{S14}$$

with $\psi = (p, v, M_1, -\partial_t M_1, M_2, -\partial_t M_2)^T$. Since now the modulation period becomes

$2T_m$, and there are 3 different phases in one modulation period: A, B and C, corresponding to resonant strength pairs $(a_{1A}, a_{2A}), (a_{1B}, a_{2B})$ and (a_{1C}, a_{2C}). The modulation cycle follows the ABAC sequence, with each phase occupying $T_m/2$ giving rise to the Floquet modes: $\psi(t+2T_m) = e^{-i\widehat{\omega}_C T_m/2} e^{-i\widehat{\omega}_A T_m/2} e^{-i\widehat{\omega}_B T_m/2} e^{-i\widehat{\omega}_A T_m/2}\,\psi(t)$. The band structure can be obtained by solving the secular equation:

$$\det\left[e^{-2i\Omega T_m} I_6 - e^{-i\widehat{\omega}_C T_m/2} e^{-i\widehat{\omega}_A T_m/2} e^{-i\widehat{\omega}_B T_m/2} e^{-i\widehat{\omega}_A T_m/2}\right] = 0. \qquad \text{(S15)}$$

Supplementary Figs. 5c and 5d in the main text plotted the band structure of phononic time crystal according to Eq.(S15) with modulation cycle following the temporal supercell ABAC sequence.
