# Supplementary material for: Temporal super-cell engineering and acoustic amplification in dispersive phononic time crystals
Source: Nat Commun. 2026 Jun 2;17:7079. doi: 10.1038/s41467-026-73459-5 (PMC13392367; doi:10.1038/s41467-026-73459-5)
Supplement: Supplementary file 2 — Transparent Peer Review file [file 41467_2026_73459_MOESM2_ESM.pdf]

nature portfolio

Peer Review File

Observation of Multiple k-gaps and Acoustic Amplification in Dispersive Phononic Time Crystals

Corresponding Author: Professor Hong-Wei Wu

This file contains all reviewer reports in order by version, followed by all author rebuttals in order by version.

Version 1:

Reviewer comments:

Reviewer #1

(Remarks to the Author)
The authors designed one-dimensional phononic time crystals by modulating the dynamic compressibility, and experimentally demonstrating the opening of a momentum gap accompanied by exponential acoustic amplification with high transmittance. They further extended their study of momentum band physics to design temporal phononic supercells, leading to the emergence of double k-gaps via momentum band folding. Although the physics of momentum gaps has been well established in the literature for over a decade, the authors have taken the challenge of experimentally observing this phenomenon in acoustics, an achievement that, to the best of my knowledge, is reported for the first time. I believe their platform could open new avenues for sound manipulation and enrich the field of acoustic wave propagation. I recommend publication in Nature Communications, provided that the authors address the following questions and comments:
• The authors state that the case in Fig. 1d is non-dispersive. Since dispersion refers to the relationship between angular frequency and momentum, could the authors clarify why this case is considered non-dispersive? In other words, what precisely do they mean by “non-dispersive” in this context?
• The description of the acoustic system composed of atoms is not sufficiently clear from Fig. 1c. Is the system an acoustic waveguide containing a series of speakers and microphones in sequence? Why are the microphone/speaker pairs arranged perpendicular to the waveguide? What are the dimensions of the waveguide, and what are the distances between the speaker–microphone pairs? It will be best if the authors could include a characterization of a single atom’s response in the Supplementary Information to provide insight into how the system behaves under temporal modulation.
• Why did the authors choose a system of nine atoms? Why not one or three?
• In lines 200–201, the authors mention a rise in transmittance for a modulation depth varying from 4.8e-2 to 10.79e-2, whereas in Fig. 3a the range appears to be less than 1.24e-3 to 4.96e-3. I do not see the correspondence between this statement and the figure. The value of delta_a mentioned in line 220 should also be confirmed.
• In lines 243–244, the authors state that bandgaps open at km/2 and km/4, while in the band structure shown in Fig. 4c, the gaps appear at k/k_m=1.5 and 3.2. Could the authors clarify this possible difference?
• In Fig. 4d, there are band discontinuities around k/km=2.5. Could the authors explain the origin of these discontinuities and the apparent separation between these k domains?

Other remarks
• In line 143, at this stage of discussion, I would avoid stating that “the similarity confirms our ability to observe the momentum gap experimentally.” I suggest replacing “ability” with “possibility.”
• The red–blue color scale in Fig. 2d should be defined.

(Remarks on code availability)
N/A

Reviewer #2

(Remarks to the Author)
The paper investigates the behavior of an array of coupled microphones and speakers with an elaborate control scheme to

achieve "effectively time-dependent compressibility". The compressibility is further frequency dependent. The paper then goes on to investigate repercussions regarding wave propagation and, in particular, the influence of "momentum bandgaps" on amplification.

The paper is well-written. It caters to a niche audience interested in time-dependent media.

I think we can agree that, theory-wise, nothing major is contributed here. Experimentally, several systems have achieved "effectively" time dependent properties using elaborate magnetic, piezoelectric, or even sensor-based couplings. Thus, I fail to see the groundbreaking novelty expected of a nature com paper. If there is a technological breakthrough pertaining to a *specific genuine* technological application, then the authors are invited to make that apparent.

(Remarks on code availability)

Reviewer #3

(Remarks to the Author)
This manuscript reports experimental observation of momentum band gaps (k-gaps) for sound transmitting through an air-filled waveguide with time-modulated compressibility. Enhanced transmission is observed for sound frequencies fitting the momentum gaps. The authors claim relatively strong modulation of air compressibility that provides k-gaps opening at relatively low frequency of modulation. However, the modulation of compressibility was not physically achieved but simulated by so-called meta-atom unit consisting of a microphone and a speaker generated artificial acoustic field which effectively corresponds to a medium with time-dependent compressibility. This method of synthetic sound modulation was proposed in the previous study [32] of the same group. Realizations of spatiotemporal and time-varying frequency-dispersive effective media with the meta-atoms were reported by them in Refs. [33,34]. The results reported in the current manuscript lack the novelty and significance to justify publication in Nature Communications. Also, there are several serious issues with the interpretation of the results obtained.

1. The demonstrated time crystals really do not represent a homogeneous medium. The meta-atoms by themselves serve as periodic scatterers, even if they are distributed along the waveguide boundary, i.e., the waveguide should be considered as a periodic spatiotemporal 1D structure. In Eq. (2) compressibility depends on x and t, as it should be. However, in the line 106 following this equation the authors state that the “medium is homogenous and infinite along the x direction.” Both these properties are questionable. The homogeneity is broken by 9 meta-atoms and the length of the sample is only 18 cm. For sound transversing the air waveguide with modulation frequency 8.4 kHz this geometrical length corresponds to only 4 full temporal unit cells. For modulation frequency 6.6 kHz the temporal length of the structure is only 3 periods. Therefore, the proposed time-modulated media can be hardly considered as Floquet crystals. The band structures presented in Figs. 1 and 4 were calculated for infinite Floquet crystals. The k-gaps presented in Figs. 1 and 4 are quite narrow. It is unlikely that such narrow gaps can be resolved in the transmission spectra of time crystals containing only 3 and 4 periods. Lack of time periodicity is probably the reason for pure agreement between theory and experiment in the transmission spectra.

2. The explanation of the proposed method of temporal modulation is very brief and unclear. Without reading Ref. [32] it is impossible to understand that the proposed method of temporal modulation of air compressibility is a digital simulation.

3. Authors discuss some results without proper explanation under what conditions they were obtained. For instance, Fig. 2d shows phase dependence of the transmission peak. However, at the figures of same block (Fig. 2c, Fig. 3a-b) there is no information about the phase.

4. The text on pp. 11-12 is confusing. The authors mention “two distinct k-gaps emerge around k_m/2 (light pink region) and k_m/4 (light cyan region)” in Fig. 4c. However, in Fig. 4c the k-gaps appear near 3/2k_m and 3k_m. The second band gap (at 3k_m) correspond to zero frequency. It is not clear how it may be experimentally observed. If this gap originates from unfolding the band structure, the authors must mention what quasi-frequency it corresponds to. The text on pp. 11-12 must be carefully revised since there the authors refer to gaps instead of resonances and vice versa. The same problem arises with the positions of the k-gaps which are given in terms of frequencies instead of wave vectors.

5. The supplements 2 and 4 are not mentioned in the main text.

(Remarks on code availability)

Reviewer #4

(Remarks to the Author)
I co-reviewed this manuscript with one of the reviewers who provided the listed reports. This is part of the Nature Communications initiative to facilitate training in peer review and to provide appropriate recognition for Early Career Researchers who co-review manuscripts.

(Remarks on code availability)

Version 2:

Reviewer comments:

Reviewer #1

(Remarks to the Author)
The authors have rigorously addressed my concerns.

(Remarks on code availability)
N/A

Reviewer #3

(Remarks to the Author)
The authors have made appropriate revisions to the manuscript, and most of the questions and comments have been adequately addressed in the response letter. The revised version is technically more sound and clearer in its presentation. However, several concerns regarding the reported results remain.
In particular, the simulated transmittance spectrum in Fig. 2c appears to have been modified achieving better agreement with the experimental data, but this change is not explicitly discussed. Furthermore, the origin of the discrepancies between theory and experiment in the transmittance and reflectance spectra shown in Figs. 5e and 5f remains unclear and should be clarified.
Regarding novelty the authors claim in their response letter, the concept of employing a supercell with perturbations to open additional band gaps has been well established in the literature (see, for example, “Degeneracy analysis for a supercell of a photonic crystal and its application to the creation of band gaps,” Phys. Rev. E 67, 026612, 2003). The concept of a temporal supercell has also been explored in prior work (e.g., “Topological wave phenomena in photonic time quasicrystals,” Phys. Rev. B 111, 125421, 2025).
Overall, while the manuscript presents solid and publishable technical work, the level of novelty and significance does not appear sufficient to justify publication in Nature Communications.

(Remarks on code availability)

Reviewer #4

(Remarks to the Author)
I co-reviewed this manuscript with one of the reviewers who provided the listed reports. This is part of the Nature Communications initiative to facilitate training in peer review and to provide appropriate recognition for Early Career Researchers who co-review manuscripts.

(Remarks on code availability)

Open Access This Peer Review File is licensed under a Creative Commons Attribution 4.0 International License, which permits use, sharing, adaptation, distribution and reproduction in any medium or format, as long as you give appropriate credit to the original author(s) and the source, provide a link to the Creative Commons license, and indicate if changes were made.
In cases where reviewers are anonymous, credit should be given to 'Anonymous Referee' and the source.
The images or other third party material in this Peer Review File are included in the article's Creative Commons license, unless indicated otherwise in a credit line to the material. If material is not included in the article's Creative Commons license and your intended use is not permitted by statutory regulation or exceeds the permitted use, you will need to obtain permission directly from the copyright holder.
To view a copy of this license, visit https://creativecommons.org/licenses/by/4.0/

Responds to the reviewer's comments:

Reviewer 1:

The authors designed one-dimensional phononic time crystals by modulating the dynamic compressibility, and experimentally demonstrating the opening of a momentum gap accompanied by exponential acoustic amplification with high transmittance. They further extended their study of momentum band physics to design temporal phononic supercells, leading to the emergence of double k-gaps via momentum band folding. Although the physics of momentum gaps has been well established in the literature for over a decade, the authors have taken the challenge of experimentally observing this phenomenon in acoustics, an achievement that, to the best of my knowledge, is reported for the first time. I believe their platform could open new avenues for sound manipulation and enrich the field of acoustic wave propagation. I recommend publication in Nature Communications, provided that the authors address the following questions and comments:

Reply: We are deeply grateful for your positive comment. In this revision, we have made every effort to address all the valuable concerns and raised suggestions.

1. The authors state that the case in Fig. 1d is non-dispersive. Since dispersion refers to the relationship between angular frequency and momentum, could the authors clarify why this case is considered non-dispersive? In other words, what precisely do they mean by "non-dispersive" in this context?

Our response: We are very sorry for the unclear expression on non-dispersive in Fig. 1d of previous version. In fact, dispersion can be categorized into modal dispersion and material dispersion: the former describes the relationship between angular frequency and momentum, while the latter arises from the frequency dependence of material parameters. In our work, the dispersion refers to material dispersion. The corresponding effective compressibility $\beta(f)$ vary gently within the frequency range marked by yellow range in Fig. 1d, thus we state that the metamaterial is nondispersive around half of modulating frequency. Inspired by you, we modify the Fig.1 and directly discuss the case of dispersive phononic time crystal by temporal modulating the frequency depended compressibility, and find more plentiful band structure that

not only presents k-gap at half modulating frequency, but also hold four extra energy gaps originated from the Lorentzian resonation and its Floquet replica due to periodical modulation. The formation mechanism of momentum band structure is illustrated in Note 2 of Supplementary Materials. Thus, we modify the Fig. 1 to a new version as:

From

“ 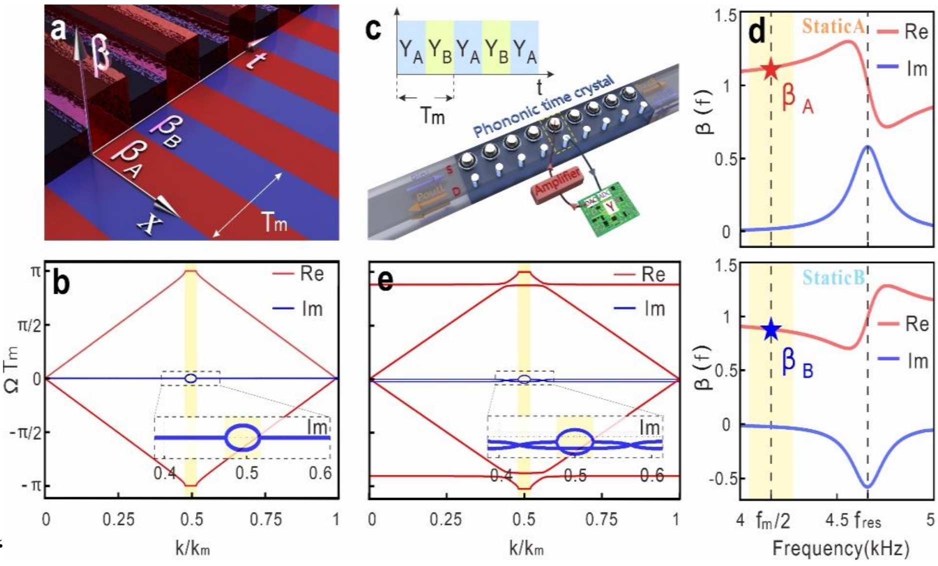 ”

to

“ 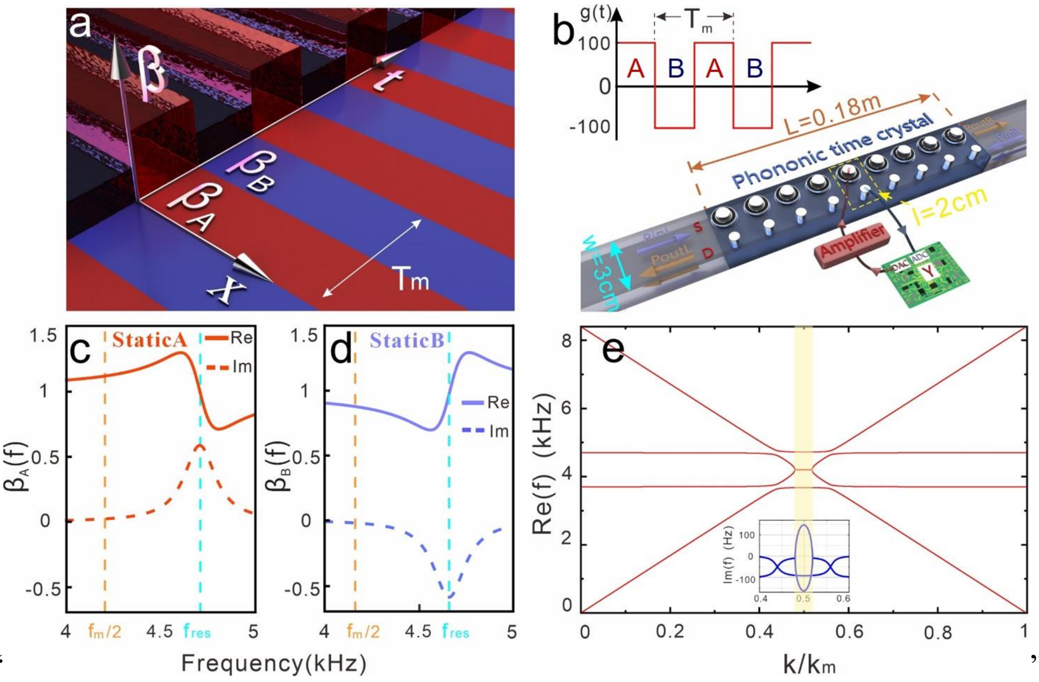 ”.

2. The description of the acoustic system composed of atoms is not sufficiently clear from Fig.

1c. Is the system an acoustic waveguide containing a series of speakers and microphones in sequence? Why are the microphone/speaker pairs arranged perpendicular to the waveguide? What are the dimensions of the waveguide, and what are the distances between the speaker–microphone pairs? It will be best if the authors could include a characterization of a single atom's response in the Supplementary Information to provide insight into how the system behaves under temporal modulation.

Our response: We are very sorry for unclear description about the acoustic system. A pair of speaker and microphone interconnected via a microcontroller is designed as a meta-atom, then we arrange 9 meta-atoms on the acoustic waveguide in sequence with subwavelength lattice length $l = 0.02m$ for forming a finite homogenous metamaterial. Each meta-atom consists of a detector and a speaker arranged perpendicular to the waveguide with cross section $3\text{cm} \times 3\text{cm}$ for eliminating the spatial phase difference along the wave propagating direction. These details have been marked in updated Fig. 1b.

Single static meta-atom with positive and negative Lorentzian response also be given in Fig. S2 of Supplementary Materials as Fig. R1. The resonating frequency is defined as 4.7kHz. The red lines and symbols represent the analytical and experimental transmission (T), the blue lines and symbols correspond to the analytical and experimental reflection (R). The yellow region indicates the forming k-gap range if temporal modulating compressibility with modulating frequency 8.4kHz between the two static case.

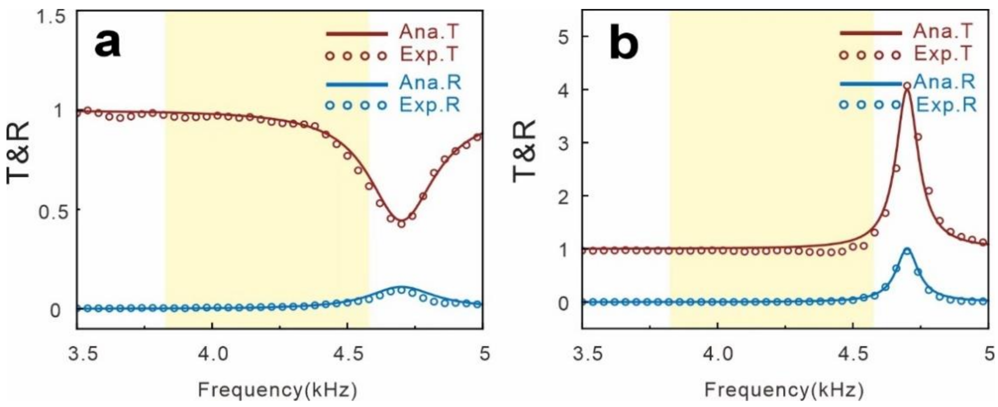

Fig. R1 the transmission (T) and reflection (R) of single static meta-atom with **a** positive and **b** negative Lorentzian response.

3. Why did the authors choose a system of nine atoms? Why not one or three?

Our response: Thank you for your valuable consideration. In our acoustic system, the 1D phononic time crystal consisted by discrete meta-atoms with subwavelength lattice distance is periodically modulated in time, while the finite spatial length along the 1D waveguide determines the effective modulated periods, and the magnitude of amplified transmission is more obvious for the longer spatial length. Theoretically, for our system, a spatial distance $d = 0.083\text{m}$ is required to support the interference between temporal refracted waves for opening the k-gap. The required distance cover 5 meta-atoms. To clarify the conclusion, we also calculate the transmission (T) and reflection (R) for different meta-atom numbers by simulation in Fig. R2. We can find that the transmission and reflection strength within the k-gap range depends on the meta-atom number N, and the phononic time crystal with more meta-atoms will present more obvious T&R. Considering the practical experiment, we construct 9 meta-atoms in our work, the T&R in Fig. R2c clearly showcase the amplified transmission. However, if we decrease the meta-atom number to three as shown in Fig. R2e, then the amplified transmission phenomenon will be difficult to observe in experiment.

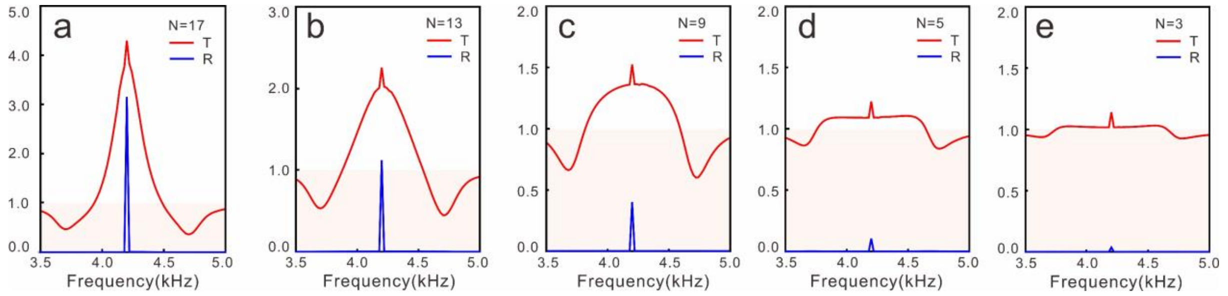

Fig. R2 Transmittance (T) and reflectance (R) of 1D phononic time crystals consisted with different meta-atom numbers N = 17, 13, 9, 5, 3.

4. In lines 200–201, the authors mention a rise in transmittance for a modulation depth varying from 4.8e-2 to 10.79e-2, whereas in Fig. 3a the range appears to be less than 1.24e-3 to 4.96e-3. I do not see the correspondence between this statement and the figure. The value of delta_a mentioned in line 220 should also be confirmed.

Our response: We are very thankful you for your meticulous inspection. It is a mistake in Fig. 3a and 3b due to our carelessness, we have check and modify the Figure 3 in the updated version, as

“

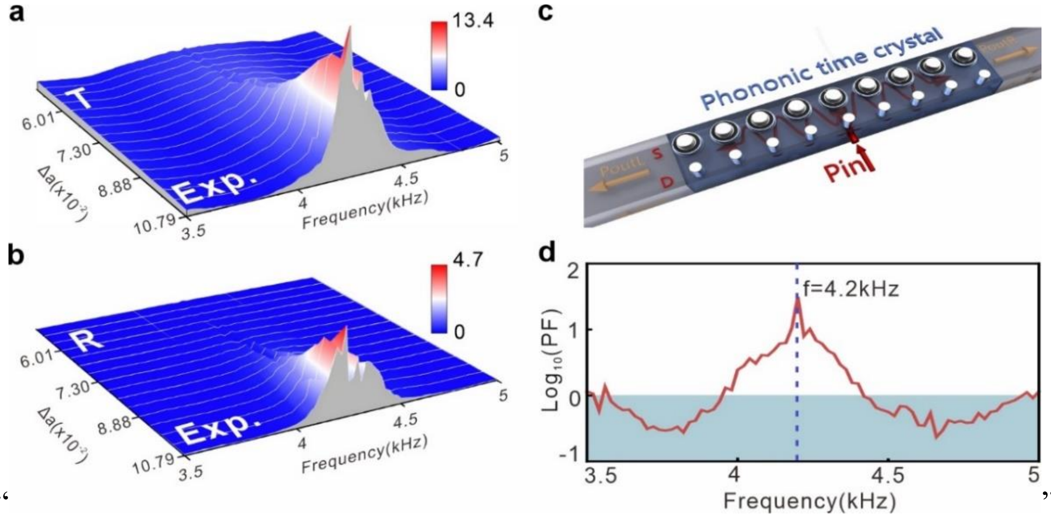

”.

5. In lines 243–244, the authors state that bandgaps open at km/2 and km/4, while in the band structure shown in Fig. 4c, the gaps appear at k/k_m=1.5 and 3.2. Could the authors clarify this possible difference?

Our response: We are very sorry for the errors in previous Fig. 4 caused by a lack of in-depth understanding. In the updated version, we check and modify the calculation of momentum band structure with supercell “ABAC”, then a 6 by 6 eigenvalue problem is given Eq. (S14) in Supplementary Materials, and the band structure can be obtained by Eq. (S15). The updated real and imaginary part of complex band structure are presented in Fig. 5c and 5d of updated manuscript or Fig. R3. The updated band structure showcases that the new k-gaps are opened at $k = \frac{1}{4}k_m$ and $k = \frac{3}{4}k_m$ due to the band folding with temporal supercell “ABAC”, except the inherent k-gap located at $\frac{1}{2}k_m$, we name them as $\frac{1}{4}k - gap$, $\frac{3}{4}k - gap$ and $\frac{1}{2}k - gap$. To clarify the momentum band folding process, we also add a band folding figure for the unit cell is expanded to supercell, as:

“
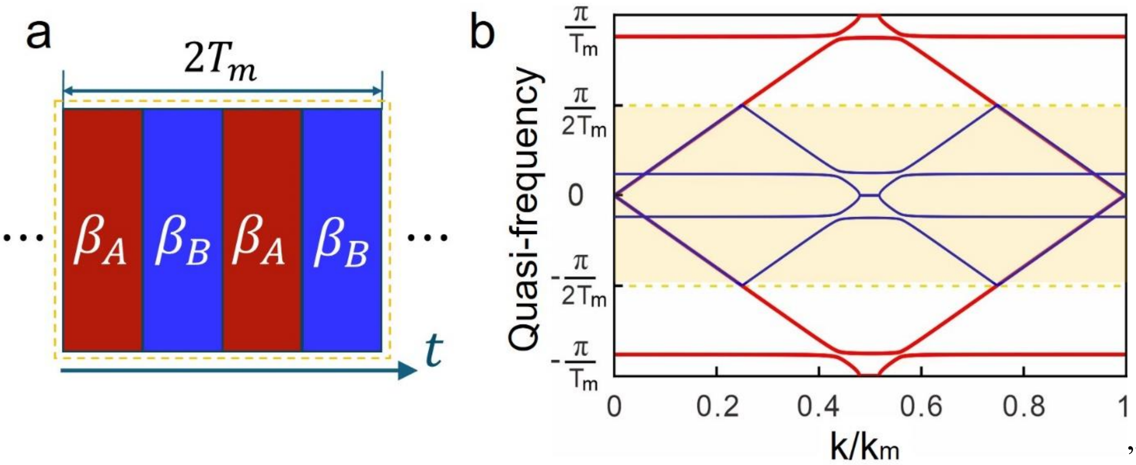

”

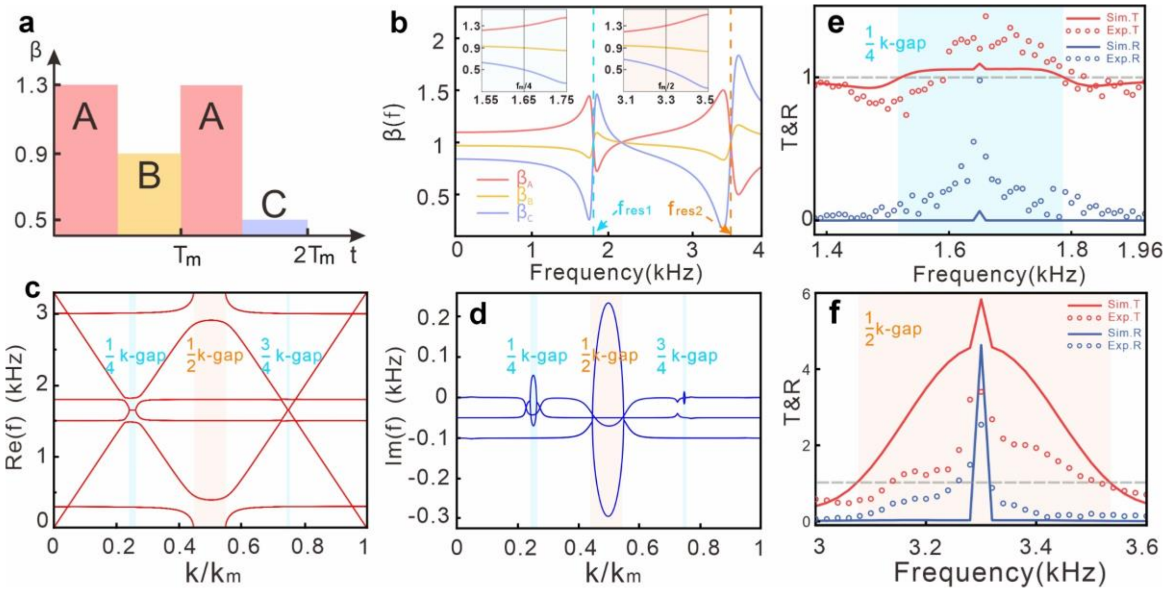

Fig. R3 **a** Conceptual picture of quasi-phononic time crystal with ABAC sequence modulation. **b** Static compressibility for different configurations A (β_A), B (β_B), C (β_C) with two resonations, the vertical cyan and orange dashed lines marked the resonating frequencies f_{res1} and f_{res2}, the top inserts shows the compressibility around the $f_m/4$ and $f_m/2$. **c**, **d** Band structure of quasi-phononic time crystal with temporal super-cell in **a** for the real and imaginary part, the bright cyan and yellow regions correspond to the 1/4 k-gap, 1/2 k-gap and 3/4 k-gap. Experimental and simulated results of transmittance and reflectance spectra around the 1/4 k-gap **e** and 1/2 k-gap **f** region.

6. In Fig. 4d, there are band discontinuities around k/km=2.5. Could the authors explain the origin of these discontinuities and the apparent separation between these k domains?

Our response: Thank you for your in-depth consideration. There are no discontinuities in the band structure, we have updated the full picture of band structure in Fig. R3 and Fig. 5 of the updated manuscript.

7. In line 143, at this stage of discussion, I would avoid stating that “the similarity confirms our ability to observe the momentum gap experimentally.” I suggest replacing “ability” with “possibility.”

Our response: Thank you for your scrupulous check and valuable suggestion. We have modified the description in updated version.

8. The red–blue color scale in Fig. 2d should be defined.

Our response: Thank you very much for your reminder, in Fig. 2d the scale of red-blue color corresponds to the phase difference $\Delta\varphi$ from 0 to 2π as label on the coordinate axis. We have updated the Fig. 2.

Reviewer 2:

The paper investigates the behavior of an array of coupled microphones and speakers with an elaborate control scheme to achieve "effectively time-dependent compressibility". The compressibility is further frequency dependent. The paper then goes on to investigate repercussions regarding wave propagation and, in particular, the influence of "momentum bandgaps" on amplification.

The paper is well-written. It caters to a niche audience interested in time-dependent media.

I think we can agree that, theory-wise, nothing major is contributed here. Experimentally, several systems have achieved "effectively" time dependent properties using elaborate magnetic, piezoelectric, or even sensor-based couplings. Thus, I fail to see the groundbreaking novelty expected of a nature com paper. If there is a technological breakthrough pertaining to a *specific genuine* technological application, then the authors are invited to make that apparent.

Reply: Thanks for your comment. In our work, we have two contributions in the field of Floquet time crystals.

In terms of theory: We propose the momentum band folding based on temporal supercell, and the multiple k-gaps can be observed not only in theoretical calculation but also experimental measurement by amplified transmission, which is first proposed in Floquet time crystals regardless in optics, acoustics, or other classical waves. It is well known that the energy band

folding and reopen gap plays an important role in spatial crystals for topological insulator, bound state in continuum, and so on. Thus we have reasons to believe that the momentum band folding may be a fundamental footstone for investigating the temporal topological insulator and other novel phenomena.

In terms of experiment: We agree with your statement that "several systems have achieved "effectively" time dependent properties using elaborate magnetic, piezoelectric, or even sensor-based couplings". However, these modulating methods such as elaborate magnetic, piezoelectric, or sensor-based couplings only effective for infrasound realizing 2-times higher than the working frequency as references [*Phys. Rev. Lett.* **121**, 194301 (2018), *Phys. Rev. Lett.* **122**, 124301 (2019), *Phys. Rev. Lett.* **133**, 077201 (2024), *Nat. Commun.* **16**, 9520 (2025)], while these methods are helpless in the face of audible sounds with high working frequency. In this work, we construct an acoustic time crystal with discrete resonating meta-atoms loading onto a one-dimensional acoustic waveguide to synthesize homogenous dispersive time-varying metamaterials by temporally modulating effective compressibility. It worthy to note that the well-designed temporal metamaterial can effectively modulate material parameters with 2-times higher than the working frequency of audible sound. With our experiment platform, we first report the experimental realization of momentum band folding and observe multiple *k*-gaps for airborne sound.

Reviewer 3:

This manuscript reports experimental observation of momentum band gaps (k-gaps) for sound transmitting through an air-filled waveguide with time-modulated compressibility. Enhanced transmission is observed for sound frequencies fitting the momentum gaps. The authors claim relatively strong modulation of air compressibility that provides k-gaps opening at relatively low frequency of modulation. However, the modulation of compressibility was not physically achieved but simulated by so-called meta-atom unit consisting of a microphone and a speaker generated artificial acoustic field which effectively corresponds to a medium with time-dependent compressibility. This method of synthetic sound modulation was proposed in the previous study [32] of the same group. Realizations of spatiotemporal and time-varying frequency-dispersive effective media with the meta-atoms were reported by them in Refs. [33, 34]. The results reported in the current manuscript lack the novelty and significance to justify

publication in Nature Communications. Also, there are several serious issues with the interpretation of the results obtained.

Reply: Thank you for your in-depth consideration. Indeed, the compressibility of metamaterials is temporally modulated by meta-atom units rather than physical structure in our work. The reason is that the temporal modulation of physical structure is seriously limited by the frictional loss of the mechanical motion, resulting to slow modulating speed and small modulating depth. For example, C. Shen *et.al* propose nonreciprocal acoustic transmission in space-time modulated coupled resonators by piston motion to change the resonating cavities volume as shown in Ref [*Phys. Rev. B* **100**, 054302 (2019)], the modulating frequency is very low comparing with the working frequency. Furthermore, a robust acoustic pumping in waveguide with dynamic boundary driven by a rotating motor also be physical observed by X. Xu *et.al* in Ref. [*Phys. Rev. Lett.* **125**, 253901 (2020)], while the speed of the dynamic modulation is confined at adiabatic evolution range.

However, to open the *k*-gap in Floquet time crystal, the high modulating frequency is required to induce the interference between 0 order and -1st order Floquet modes. This requirement is a huge challenging for conventional mechanical modulation. By contrast, the time-varying metamaterial constructed by meta-atoms holds advantages of multiple parameters modulatable, high modulating frequency, low losses and high efficiency. In our previous works [*Phys. Rev. B* **104**, L060304, (2021), *Phys. Rev. B* **108**, 104303, (2023)], the modulating frequency can realize 7 times higher than working frequency, then we investigate the effective medium formulas of temporal metamaterial for modulating different resonating parameters.

Different from our previous works, here we focus on folding the momentum band structure and opening multiple k-gaps by introducing temporal supercell for modulating frequency is 2 times higher than working frequency. In this work, the proposed momentum band folding with temporal supercell remains rarely reported regardless in optical, acoustic, or other waves, whether in theory or in experiment.

In this revision, we have made every effort to address all the valuable concerns and raised suggestions.

1. The demonstrated time crystals really do not represent a homogeneous medium. The meta-

atoms by themselves serve as periodic scatterers, even if they are distributed along the waveguide boundary, i.e., the waveguide should be considered as a periodic spatiotemporal 1D structure. In Eq. (2) compressibility depends on x and t, as it should be. However, in the line 106 following this equation the authors state that the "medium is homogenous and infinite along the x direction." Both these properties are questionable.

Our response: Thank you very much for your thoughtful consideration and valuable comments. We are very sorry for our unclear expression. In our work, the discrete meta-atoms periodically distribute along the waveguide with subwavelength lattice length comparing with working wave length, such as the parameters: lattice length $l = 0.02m$, working wavelength around $\lambda_0 = 0.08m$. Thus, along the waveguide, we can treat the discrete meta-atom array as a spatial homogenous metamaterial with a finite length $L = Nl$, where N is the meta-atom number. To demonstrate that the discrete meta-atom array can be equivalent to a homogenous metamaterial, we calculate the transmission (T) and reflection (R) of the discrete 9 meta-atoms with the static Lorentzian response $Y(f) = \frac{ifg}{f_{res}^2 - f^2 - 2i\gamma f}$ and an effective homogenous media with $\beta(f) \approx 1 + \frac{2c_0}{ifl} Y(f)$ for same spatial length $L = 0.18m$, as shown in Figs. R4a and R4b. The parameters are selected as: resonant strength $g = 100$ Hz, resonant frequency $f_{res} = 4.7$ kHz and linewidth $\gamma = 100$ Hz, $c_0 = 343$ m/s corresponds to sound speed in air. It is not to find that the T is nearly identical for the two cases, the slight difference for the R in Figs. R4a and R4b comes from the approximate formula for effective medium. Anyhow, the meta-atom array can be equivalent as a spatially homogenous material ($l \ll \lambda_0$) with finite spatial length L.

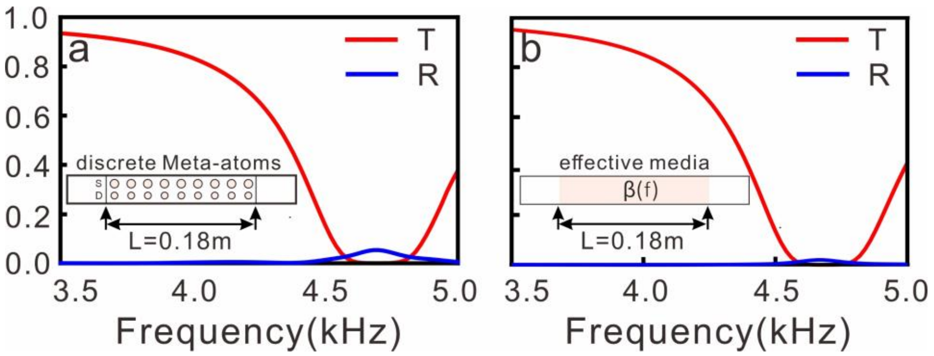

Fig. R4 Transmission (T) and reflection (R) of discrete meta-atom array and effective media with same spatial length.

The homogeneity is broken by 9 meta-atoms and the length of the sample is only 18 cm. For sound transversing the air waveguide with modulation frequency 8.4 kHz this geometrical length corresponds to only 4 full temporal unit cells. For modulation frequency 6.6 kHz the temporal length of the structure is only 3 periods. Therefore, the proposed time-modulated media can be hardly considered as Floquet crystals. The band structures presented in Figs. 1 and 4 were calculated for infinite Floquet crystals. The k-gaps presented in Figs. 1 and 4 are quite narrow. It is unlikely that such narrow gaps can be resolved in the transmission spectra of time crystals containing only 3 and 4 periods. Lack of time periodicity is probably the reason for pure agreement between theory and experiment in the transmission spectra.

Our response: Indeed, the phononic time crystal with a finite spatial length along the 1D waveguide dictates the effective modulation period. This also implies that the amplified transmission becomes more pronounced for phononic time crystals with a longer spatial length. However，the modulating period number in the spatial length $L = 0.18\text{m}$ can not simply understand as $\frac{L}{c_0 T_m} = 4.5$ for modulating frequency $f_m = 8.4\text{kHz}$. It well known that the k-gap emerges from the interference of the multiple pathways in the first few orders. To clarify the physical picture, we plot $c_0 t - x$ diagram using the nondispersive compressibility $\beta_A = 1.2$ and $\beta_B = 0.8$ corresponding to the value of working frequency 4.2kHz as shown in Fig. R5. A wave packet is injected into the phononic time crystal with phase "A" from the left end, when the packet propagates over $T_m/2$, it will meet the first temporal interface, and generates spatial distance $d_A = \frac{c_0}{n_A}\frac{T_m}{2} = 0.019\text{m}$, and $h = \frac{c_0 T_m}{2} = 0.02\text{m}$. Crossing the interface, the packet will splits into forward refracted wave packet and backward reflected wave packet. The temporal reflection and transmission coefficient from phase "A" to "B" can be written as $|r|_A^B = \frac{1}{2}\left(\frac{\beta_A}{\beta_B} - \frac{n_A}{n_B}\right) = 0.14$ and $|r|_A^B = \frac{1}{2}\left(\frac{\beta_A}{\beta_B} + \frac{n_A}{n_B}\right) = 1.36$ according to temporal Snell's law, n_A and n_B are the refractive index. The incident, reflected and refracted angle satisfy the relation: $\frac{tan\theta_A^i}{tan\theta_B^t} = \frac{d_A}{d_B} = \frac{n_B}{n_A}$, and $\theta_{2t} = \theta_{2r}$, here $d_B = \frac{c_0}{n_B}\frac{T_m}{2} = 0.023\text{m}$. These angles approximate 45° due to $n_A \approx 1.1$ and $n_B \approx 0.9$. At second temporal interface, the forward refracted wave packet and backward reflected wave packet will further split into more wave

packets along forward and backward direction. When the packet propagate from phase “B” to “A”, $|r|_B^A = 0.075$ and $|t|_B^A = 0.74$. The leftmost reflect wave will go out from the left spatial boundary with very small reflection.

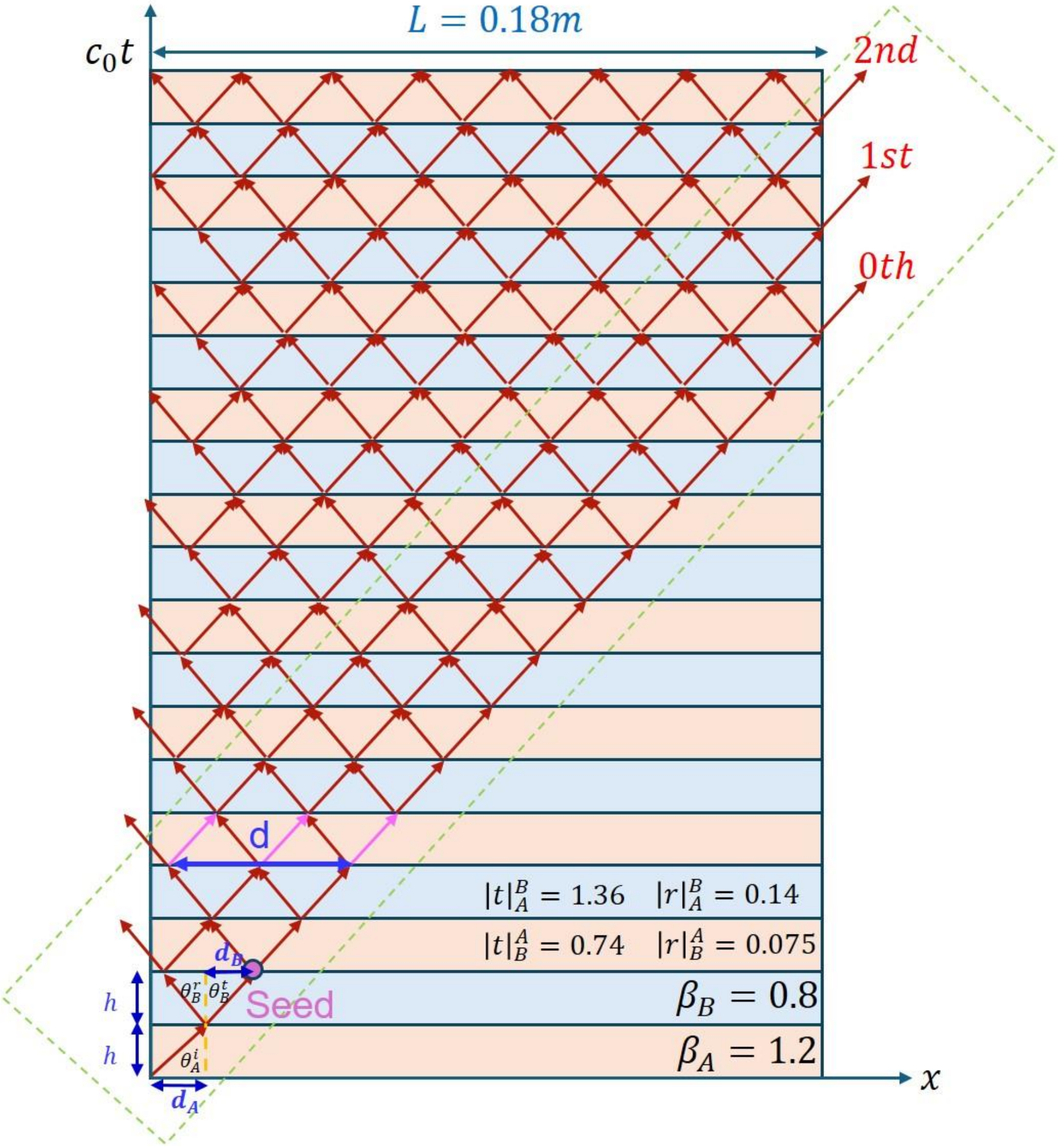

Fig. R5 temporal reflection and refraction cross over multiple temporal interfaces.

Particularly, the rightmost point marked by “Seed” will induce three refracted waves as shown as pink arrows over a modulating period to interference each other for open the k-gap. The three arrows correspond to the 0-th, 1-st and 2-nd temporal refracted wave, which come from the waves experienced 0 times, 1 time, 2 times temporal reflection. That is to say, we need

at least spatial length $d = 2(d_A + d_B) = 0.083\text{m}$ to support them interference to get k-gap along the beam before exit. Now, our 9 meta-atoms cover the spatial length $L = 0.18$m, which is two times of the required distance. Thus, the k-gap is opened absolutely. In fact, the open of the k-gap only need 5 meta-atoms due to $\frac{d}{l} = 4.15$.

To clarify above conclusion, we calculate the transmission and reflection of phononic time crystal for different meta-atom numbers in Fig. R6. We can find that the transmission and reflection magnitude within the k-gap depends on the meta-atom numbers, and more meta-atoms more obvious T&R. Considering the practical experiment, we construct 9 meta-atoms in our work, the T&R in Fig. R5c clearly presents the amplified transmission, which also be experiment observed in Fig. 2c of main text. Particularly, for case of 5 meta-atoms, the amplified transmission is also obvious because the k-gap has been opened.

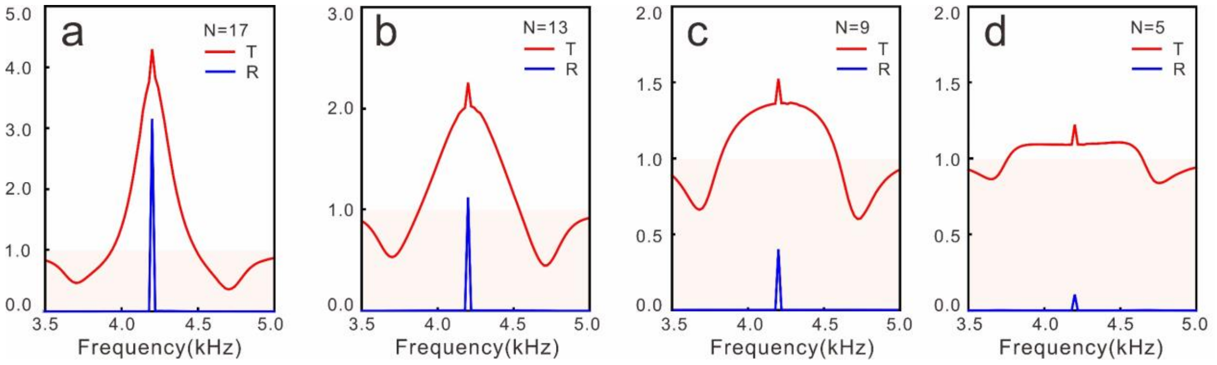

Fig. R6 Transmittance (T) and reflectance (R) of 1D phononic time crystals consisted with different meta-atom numbers $N = 17, 13, 9, 5$.

Furthermore, we can compare the momentum band structure showcased in Fig. 1e and the transmission spectrum in Fig. 2c of updated manuscript. The transmission spectrum holds the band structure characteristic that not only presents an obvious k-gap around half modulating frequency 4.2kHz from 3.8kHz to 4.6kHz, but also presents ω-gaps at resonating frequency 4.7kHz and its Floquet replica 3.7kHz (the formation of the momentum band structure is illustrated in Note 2 of Supplementary Materials). Thus, we can observe two transmission dips located at 4.7kHz and 3.7kHz always in Figs. 2c and 2d of main text or Fig. R6c. It also to say that the Floquet crystal with finite spatial length presents similar transmission spectrum comparing with one of infinite length, the difference lies merely in the transmission magnitude.

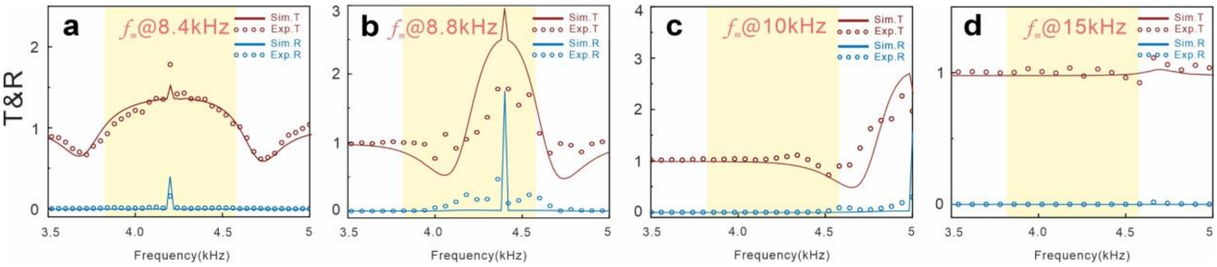

Fig. R7 The transmission and reflection of 1D phononic time crystal for different modulating frequency **a** 8.4kHz, **b** 8.8kHz, **c** 10kHz, **d** 15kHz by simulation and experiment.

To further demonstrate that the amplified transmission originates in the k-gap around the half modulating frequency, we increase the modulating frequency from 8.4kHz to 15kHz, and keep the resonating frequency at 4.7kHz as Fig. R7. It is not difficult to find that the amplified transmission range will moves away from original frequency range marked by yellow region with increasing the modulating frequency, and finally the transmission and reflection restore calm for $f_m = 15$kHz. The result means that the amplified transmission results from the k-gap around half of modulating frequency.

In addition, to demonstrate the k-gap originated from temporal modulating resonating strength, we also do fast Fourier transform algorithm (FFT) for the outputting signal when the frequency of inputting signal scanning from 3.5kHz to 5.0kHz in experiment. We can find that the outputting signal with same frequency as the inputting signal for closing all meta-atoms, i.e., a test experiment for air waveguide as shown in Fig. R8a. However, when we open all meta-atoms to temporally modulate the resonating strength $g(t)$ between 100Hz and -100Hz with modulating frequency 8.4kHz, it is not difficult to find that the -1st order Floquet mode will be generated together with the 0 order Floquet mode, and cross at half modulating frequency 4.2kHz, as can be seen in Fig. R8b. The result also demonstrates that the 0 and -1st order Floquet modes are coexist in our experiment and interference each other for forming k-gap.

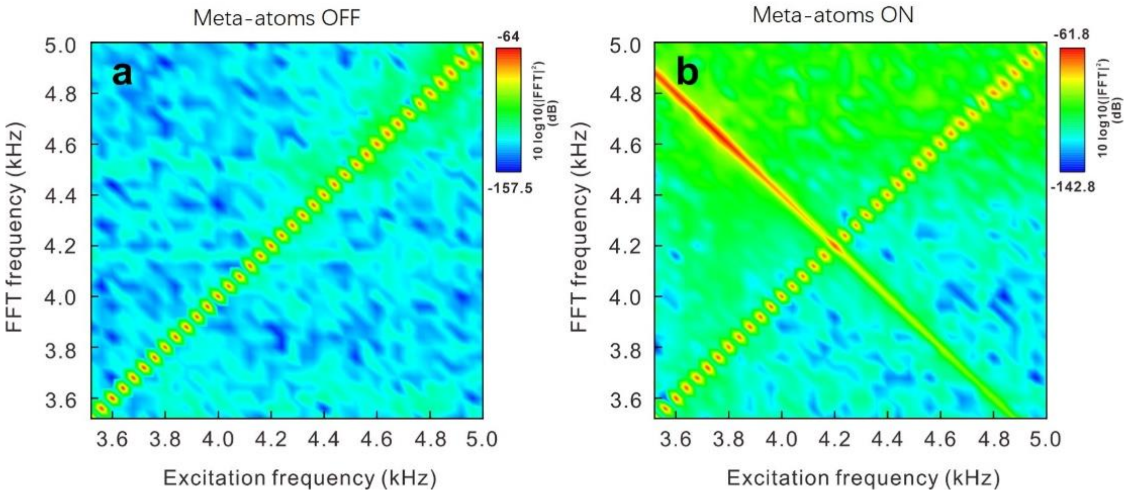

Fig. R8 The outputting signal analysis for incident signal with frequency from 3.5kHz to 5.0kHz for **a** closing meta-atoms (air waveguide) and **b** opening meta-atoms (phononic time crystal).

Although the k-gaps appears to be quite narrow in full band structure, they actually cover a frequency range of several hundred hertz. For example, the k-gap has a width from 3.8kHz to 4.6kHz for modulating frequency 8.4kHz and resonating frequency 4.7kHz. The transmission spectrum is fully capable of capturing all the characteristics of the band structure in experiments as Fig. 2.

2. The explanation of the proposed method of temporal modulation is very brief and unclear. Without reading Ref. [32] it is impossible to understand that the proposed method of temporal modulation of air compressibility is a digital simulation.

Our response: We apologize for the unclear description of the temporal modulation method. The operation processes for the nine meta-atoms have now been added to Note 1 in the revised Supplementary Materials.

3. Authors discuss some results without proper explanation under what conditions they were obtained. For instance, Fig. 2d shows phase dependence of the transmission peak. However, at the figures of same block (Fig. 2c, Fig. 3a-b) there is no information about the phase.

Our response: We are very sorry for the missing illustration for the phase. We have added the schematic diagram for explaining phase difference in Fig. 2c, and add the illustration of $\Delta\varphi = 0$ in the descriptions of the Fig. 2c, Figs. 3a-3b as well as Fig. 5 in the updated manuscript. The phase different is denoted in inert of Fig. 2c as:

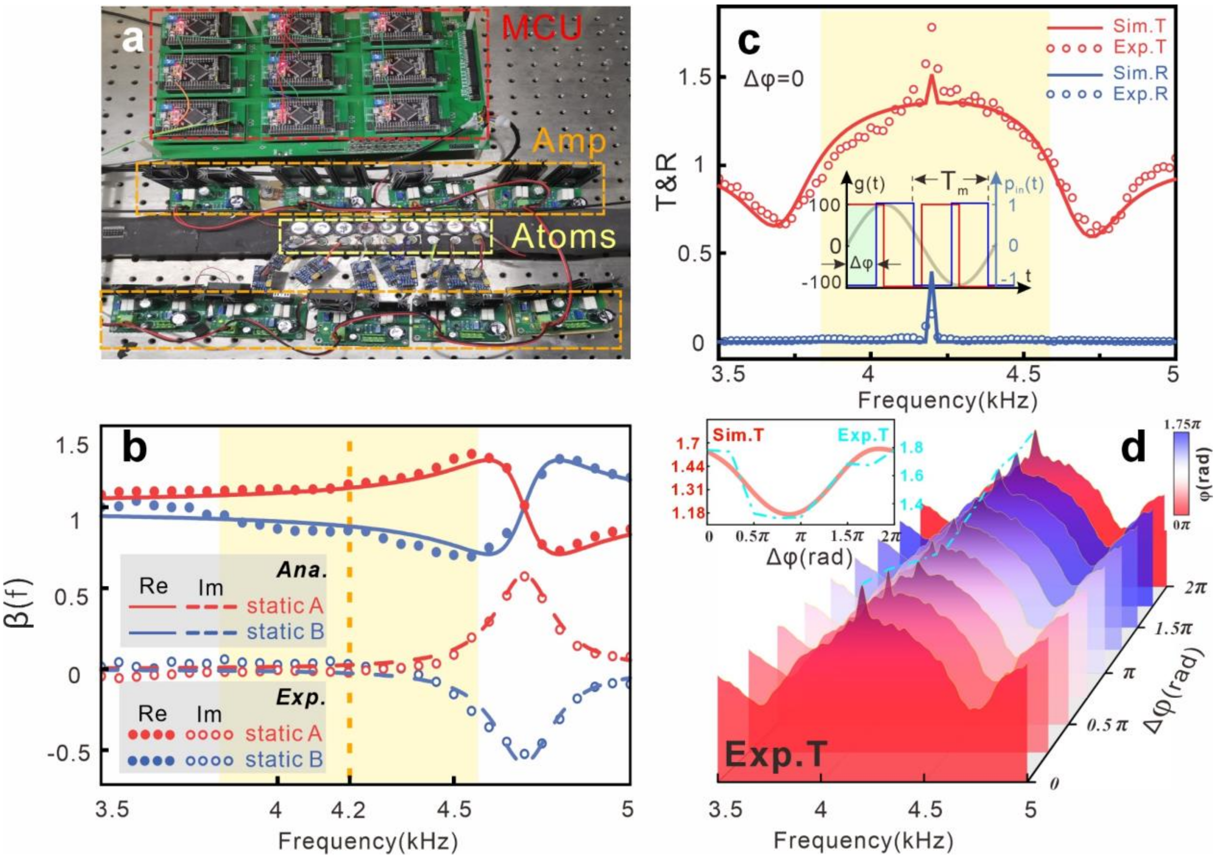

4. The text on pp. 11-12 is confusing. The authors mention "two distinct k-gaps emerge around k_m/2 (light pink region) and k_m/4 (light cyan region)" in Fig. 4c. However, in Fig. 4c the k-gaps appear near 3/2k_m and 3k_m. The second band gap (at 3k_m) correspond to zero frequency. It is not clear how it may be experimentally observed. If this gap originates from unfolding the band structure, the authors must mention what quasi-frequency it corresponds to. The text on pp. 11-12 must be carefully revised since there the authors refer to gaps instead of resonances and vice versa. The same problem arises with the positions of the k-gaps which are given in terms of frequencies instead of wave vectors.

Our response: We are very sorry for the errors in previous Fig. 4 due to a lack of in-depth understanding in previous stage. In updated version, we check and modify the calculation of momentum band structure with supercell "ABAC", then a 6 by 6 eigenvalue problem is given Eq. (S14) in Supplementary Materials, and the band structure can be obtained by Eq. (S15). The updated real and imaginary part of complex band structure are presented in Fig. 5c and 5d (also seen Fig. R9). The updated band structure showcases that the new k-gaps are open at $k = \frac{1}{4}k_m$ and $k = \frac{3}{4}k_m$ due to the band folding with temporal supercell "ABAC", except the

inherent k-gap located at $\frac{1}{2}k_m$, named as $\frac{1}{4}k-gap$, $\frac{3}{4}k-gap$ and $\frac{1}{2}k-gap$.

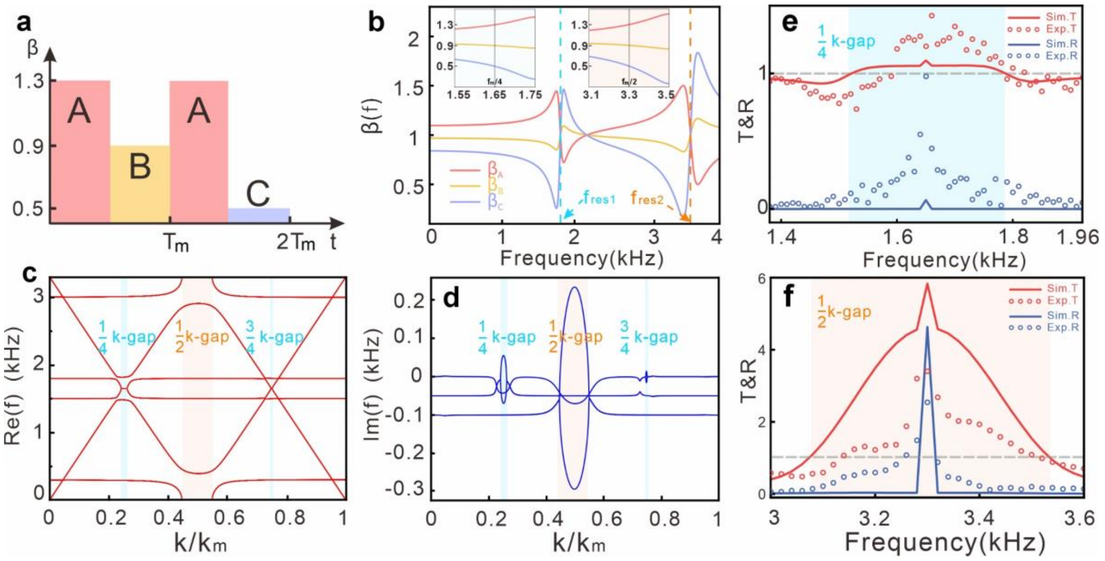

Fig. R9 **a** Conceptual picture of quasi-phononic time crystal with ABAC sequence modulation. **b** Static compressibility for different configurations A (β_A), B (β_B), C (β_C) with two resonations, the vertical cyan and orange dashed lines marked the resonating frequencies f_{res1} and f_{res2}, the top inserts shows the compressibility around the $f_m/4$ and $f_m/2$. **c**, **d** Band structure of quasi-phononic time crystal with temporal super-cell in **a** for the real and imaginary part, the bright cyan and yellow regions correspond to the 1/4 k-gap, 1/2 k-gap and 3/4 k-gap. Experimental and simulated results of transmittance and reflectance spectra around the 1/4 k-gap **e** and 1/2 k-gap **f** region.

The $\frac{1}{4}$ k-gap and $\frac{3}{4}$ k-gap come from the band folding and reopen as plotted in cyan region, the $\frac{1}{4}$ k-gap has a gap width from $0.242k_m$ to $0.262k_m$, which corresponds to frequency range from 1.57kHz to 1.73kHz around $\frac{f_m}{4}=1.65$kHz. For the selection of parameters in compressibility, the $\frac{3}{4}$ k-gap at $k=0.75k_m$ is too narrow to observe in experiment. The $\frac{1}{2}$ k-gap with a width from $0.45k_m$ to $0.55k_m$ as orange region corresponds to the frequency range from 2.97kHz to 3.63kHz around $\frac{f_m}{2}=3.3$kHz. Due to the high flexibility of our time-varying metamaterials in tailoring resonating responses as discussion in Note 1 of Supplementary Materials, we experimentally implement the modulation for dispersive media with the compressibility shown in Fig. 5b at the phase difference $\Delta\varphi=0$. Figure R9e shows the transmissivity and reflectivity at the frequency range around 1.65kHz corresponding to the $\frac{1}{4}$ k-gap. The experiment results confirm the open of the band folding induced k-gap, as

indicated by transmission spectrum greater than 1. Here, the amplified transmission is hard to observe around $\frac{3}{4}$ k-gap due to the narrower k-gap as shown in Fig. R9c and R9d. Furthermore, a similar high transmission spectrum is also observed at the second k-gap near $k_m/2$, as shown in Fig. R9f. The stronger transmission in the $\frac{1}{2}$ k-gap arises because the finite spatial length L = 0.18 m of phononic time crystal covers more modulating periods for higher operational frequencies.

To clarify the momentum band folding process, we also add a momentum folding figure (added Fig. 4 in revised manuscript) for the unit cell expends to supercell, as:

"

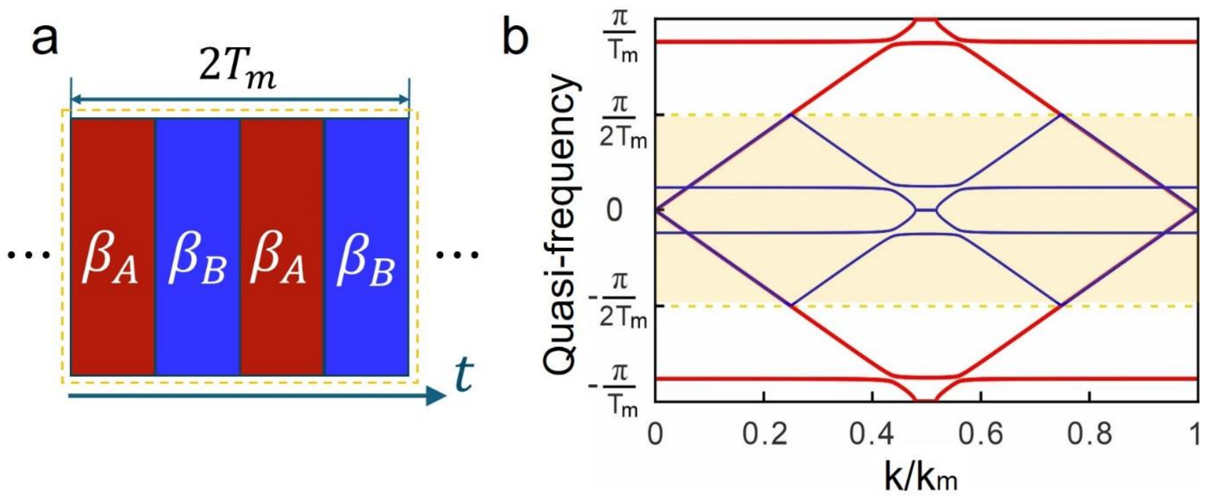

"

5. The supplements 2 and 4 are not mentioned in the main text.

Our response: We are very sorry for our carelessness. We have added denotes of supplementary materials at relevant position in main text.

Reviewer 4:

I co-reviewed this manuscript with one of the reviewers who provided the listed reports. This is part of the Nature Communications initiative to facilitate training in peer review and to provide appropriate recognition for Early Career Researchers who co-review manuscripts.

This email has been sent through the Springer Nature Tracking System NY-610A-NPG&MTS

Our response: Thank you very much for your comments on our article. We have carefully

responded the reviewer's comments of above reviewers.

Responds to the reviewer's comments:

Reviewer 1:

The authors have rigorously addressed my concerns.

Reply: We sincerely appreciate your positive feedback on our revised version.

Reviewer 3:

The authors have made appropriate revisions to the manuscript, and most of the questions and comments have been adequately addressed in the response letter. The revised version is technically more sound and clearer in its presentation. However, several concerns regarding the reported results remain.

In particular, the simulated transmittance spectrum in Fig. 2c appears to have been modified achieving better agreement with the experimental data, but this change is not explicitly discussed. Furthermore, the origin of the discrepancies between theory and experiment in the transmittance and reflectance spectra shown in Figs. 5e and 5f remains unclear and should be clarified.

Reply:

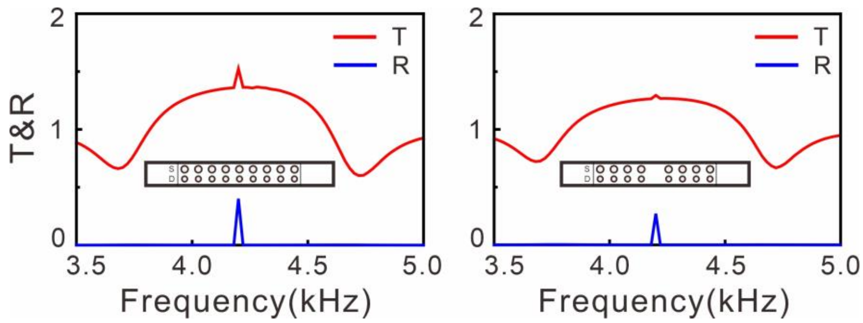

Fig. R1 Simulated T&R for defect-free (left insert) and defective (right insert) phononic time crystal.

We are very grateful to you for recognizing and approving our previous revisions. Furthermore, we sincerely apologize for the lack of explicit explanation for the discrepancies between the simulation and experimental results in Fig. 2c and Figs. 5e and 5f. In updated Fig. 2, the better simulated result is attributed to the activation of a middle meta-atom in the previous simulation, which was omitted in original revision. However, in Figs. 5e and 5f, the discrepancy between the simulated and experimental results mainly arises

from the small compressibility contrast between Phase B and Phase C (Fig. 5a). This leads to a narrow $\frac{1}{4}$k-gap width, which increases the difficulty of achieving a good match between the experimental and simulated results. In updated Fig. 5, we increase the compressibility contrast between static phase B and C, and effectively broadening the $\frac{1}{4}$k-gap width. The re-measured experimental results show much better agreement with the simulated counterparts. These detail explanations and modifications are presented as follows:

1. Explanation for the changes in Figure 2

Indeed, the simulated transmittance spectrum in Fig. 2c has been modified in last revision. The reason is that an oversight was identified in the original simulation when we prepared the previous response letter. A middle meta-atom was non-functional due to our omission in the simulation, as shown in the right inset of Fig. R1, and resulted in the T&R with low strength compared to the T&R of the defect-free one, as shown in the left plot of Fig. R1. Notably, the correct simulation result agrees well with the experiment. Since it is about correcting errors and presenting the correct results, we do not include additional discussion for this modification in the main text. We are very sorry for causing you any confusion.

2. Clarifying the origin of discrepancies between simulation and experiment in Figures 5e and 5f

Thank you very much for pointing out the discrepancies between simulation and experiment in Figs. 5e and 5f. In the previous version, the mismatch between the simulated and experimental results originates from the small compressibility contrast between static Phases B and C. This leads to a narrow $\frac{1}{4}$k-gap width. The correspondingly weak amplified transmission further increases the difficulty for the experimental results to match the numerical predictions, as presented in Fig. R2.

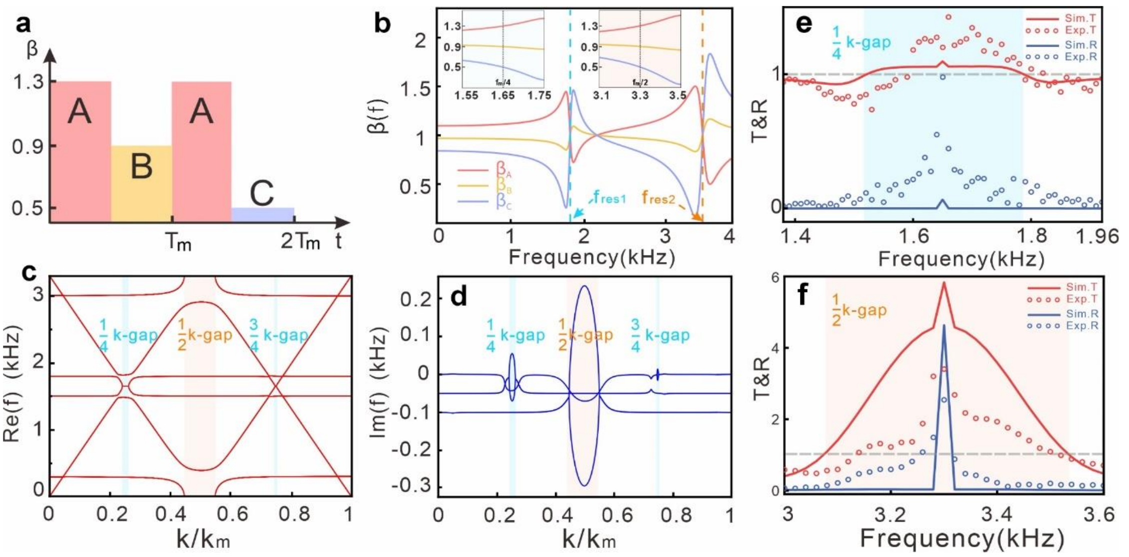

Fig. R2 Original figure 5 in last version. The static compressibility values of phases "A", "B" and "C" are obtained by setting resonating strengths in Eq. (7) of main text as: $a_{1A} = 3a_{10}$, $a_{2A} = 3a_{20}$ for obtaining $\beta_A = 1.3$, $a_{1B} = -a_{10}$, $a_{2B} = -a_{20}$ for obtaining $\beta_B = 0.9$ and $a_{1C} = -5a_{10}$, $a_{2C} = -5a_{20}$ for obtaining $\beta_C = 0.5$ at $f_m/4$ and $f_m/2$, where $a_{10} = -0.013$, $a_{20} = -0.0186$.

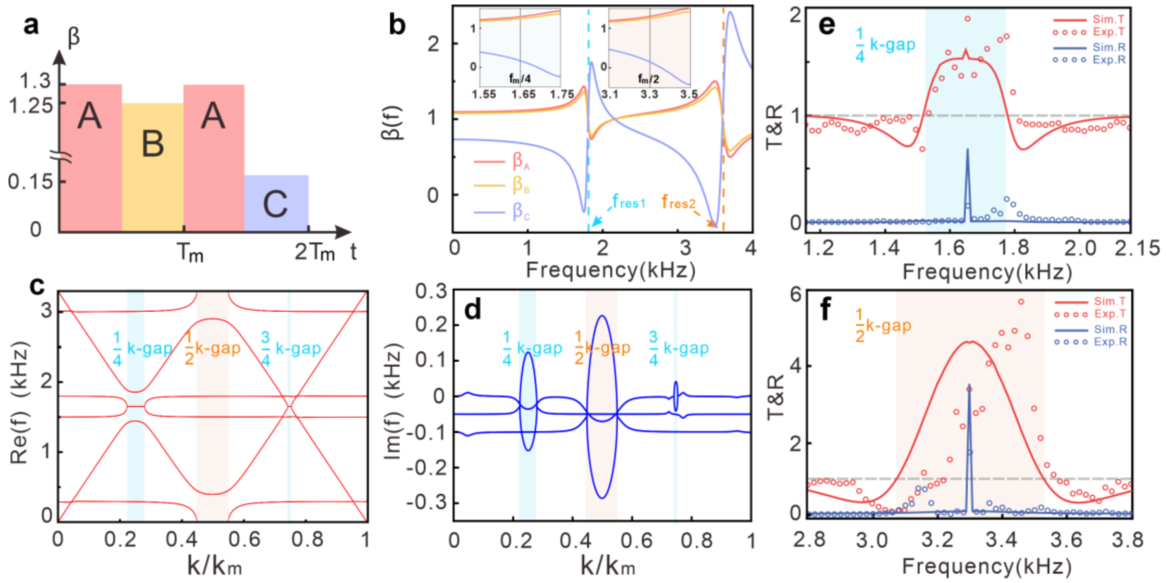

Fig. R3 Updated figure 5 in revised manuscript. The updated compressibility values of static phases "A", "B" and "C" correspond to the resonating strengths in Eq. (7) of main text as: $a_{1A} = 3a_{10}$, $a_{2A} = 3a_{20}$ for obtaining $\beta_A = 1.3$, $a_{1B} = 2.5a_{10}$, $a_{2B} = 2.5a_{20}$ for obtaining $\beta_B = 1.25$ and $a_{1C} = -8.5a_{10}$, $a_{2C} = -8.5a_{20}$ for obtaining $\beta_C = 0.15$ at $f_m/4$ and $f_m/2$, where $a_{10} = -0.013$, $a_{20} = -0.0186$.

To broaden the $\frac{1}{4}$k-gap width for easier experimental observation, we increase the compressibility contrast between static Phases B and C, as illustrated in Fig. R3a. As shown in

Figs. R3c and R3d, the $\frac{1}{4}$ k-gap width is evidently enlarged compared with the previous version. Using these optimized parameters, we re-measure the transmission and reflection (T&R) experimentally. The experimental results show much better agreement with the simulated counterparts, as presented in Figs. R3e and R3f.

Regarding novelty the authors claim in their response letter, the concept of employing a supercell with perturbations to open additional band gaps has been well established in the literature (see, for example, "Degeneracy analysis for a supercell of a photonic crystal and its application to the creation of band gaps," Phys. Rev. E 67, 026612, 2003). The concept of a temporal supercell has also been explored in prior work (e.g., "Topological wave phenomena in photonic time quasicrystals," Phys. Rev. B 111, 125421, 2025).

Overall, while the manuscript presents solid and publishable technical work, the level of novelty and significance does not appear sufficient to justify publication in Nature Communications.

Reply: Thank you very much for the insightful and constructive comments. We fully agree that both the spatial supercell strategy for opening photonic band gaps and the conceptual proposal of temporal supercells in photonic time quasicrystal have been reported in previous studies [Phys. Rev. E 67, 026612 (2003), Phys. Rev. B 111, 125421 (2025)]. However, we emphasize that **our work fundamentally differs from all prior literature in physical mechanism, band engineering dimension, and experimental realization**.

1. Fundamental Difference: Spatial Energy Band Folding vs. Temporal Momentum Band Folding

Indeed, literature [Phys. Rev. E 67, 026612 (2003)] elaborately demonstrated that the band structure of a photonic crystal with a spatial supercell exhibits intrinsic degeneracy at the Bloch Brillouin zone boundary. Such degeneracy can be lifted by breaking spatial translational symmetry, thereby generating new ω-gaps. Bloch Brillouin zone folding and the resultant emergent ω-gaps can give rise to a variety of intriguing physical phenomena. Representative examples include Brillouin zone folding-induced bound states in the continuum [Nat. Commun. 14, 2811 (2023); Sci. Adv. 7, eabk1117 (2021)], as well as topological phase transitions modulated via band inversion engineering [Nat. Phys. 9, 795 (2013); Nat. Phys. 11, 240 (2015); Phys. Rev. Lett. 117, 224301 (2016)], among others.

Nevertheless, **all these established phenomena originate from energy band folding in spatial crystals and operate only in the space domain**, rather than momentum band folding and operate in the time domain. It remains an open challenge in Floquet time crystal research how to directly fold momentum bands within the Floquet Brillouin zone and deterministically control the momentum band gap by introducing a well-defined temporal supercell, which is a fundament of the topological phase transition. **Our work resolves this key open problem.**

2. Essential Distinction: Quasicrystal-Based Temporal Supercell vs. Floquet-Modulated Temporal Supercell

The concept of a temporal supercell was explicitly introduced in literature [Phys. Rev. B 111, 125421 (2025)] to explore the temporal analog of photonic quasicrystals. Nevertheless, the supercell defined therein describes nonperiodic temporal sequences with long-range order for constructing time quasicrystals, rather than enlarging the temporal lattice to induce momentum band folding within the Floquet Brillouin zone. Accordingly, the physical motivation, structural definition, and physical application of the supercell concept differ fundamentally between the literature [Phys. Rev. B 111, 125421 (2025)] and our study.

Moreover, temporal quasicrystals lack a conventional integer scaling relation from unit cell to supercell. For this reason, the temporal supercell in literature [Phys. Rev. B 111, 125421 (2025)] is adopted merely to establish periodic boundary conditions for approximate band structure calculations and theoretical topological analysis. By contrast, the temporal supercell in our work corresponds to a well-defined integer-period extension of a periodic Floquet time crystal. As the modulation period expands, the momentum band structure is systematically folded into the Floquet Brillouin zone, and new k-gaps emerge precisely at the band-folding points by engineered breaking of temporal translational symmetry. **Notably, our experiments provide the first experimental demonstration that the newly formed 1/4 k-gap enables sound-amplifying transmission, as clearly evidenced in Fig. 5e.**

3. Core Novelty: Systematic Experimental Framework for Momentum Band Engineering in Time Crystals

In short, the temporal supercell proposed in our work provides a systematic route to engineer momentum band structures in Floquet time crystals. By extending the modulation period, it enables predictable band folding and controllable k-gap opening (e.g., the observed

1/4-k gap), beyond ad hoc temporal modulation. The resulting k-gaps directly lead to sound-amplifying transmission, demonstrating nontrivial wave phenomena enabled by momentum band engineering. Particularly, this control arises from hierarchical temporal modulation, where multiple periodicities introduced by the supercell provide structured degrees of freedom for independently tuning band folding, k-gap formation, and amplification. Such hierarchical control also enables more flexible engineering of band topology, while the temporal implementation allows in situ tuning within a single platform.

Our platform makes this experimentally accessible. To our knowledge, temporal supercells have not been demonstrated experimentally, likely due to limited control over temporal modulation. Our system enables programmable spatiotemporal modulation and realization of both fundamental and supercell-induced k-gaps, providing a practical basis for exploring more complex band and topological effects.

Taken together, the temporal supercell approach does not merely extend existing concepts, but establishes an experimental framework for momentum band engineering in Floquet systems, enabling access to both band structures, amplification regimes, and future topological phenomena that are not attainable with conventional temporal modulation schemes.

Reviewer 4:

I co-reviewed this manuscript with one of the reviewers who provided the listed reports. This is part of the Nature Communications initiative to facilitate training in peer review and to provide appropriate recognition for Early Career Researchers who co-review manuscripts.

Reply: Thank you very much for your comments on our article. We have carefully responded the reviewer’s comments of above reviewers.
